# Bridging systemic metabolic dysfunction and Alzheimer’s disease: the liver interface

**DOI:** 10.1186/s13024-025-00849-6

**Published:** 2025-05-28

**Authors:** Dan Song, Yang Li, Ling-Ling Yang, Ya-Xi Luo, Xiu-Qing Yao

**Affiliations:** 1 Department of Rehabilitation, The Second Affiliated Hospital of Chongqing Medical University, No. 74 Linjiang Road, Yuzhong District, Chongqing, 400010 China; 2 Chongqing Municipality Clinical Research Center for Geriatric Medicine, No. 76 Linjiang Road, Yuzhong District, Chongqing, 400010 China; 3 Department of Rehabilitation Therapy, Chongqing Medical University, No. 1 Medical College Road, Yuzhong District, Chongqing, 400000 China

**Keywords:** Alzheimer’s disease, Hepatokines, Liver, Metabolic disorders, Metabolism

## Abstract

Alzheimer’s disease (AD) is increasingly recognized as a systemic disorder with a substantial metabolic disorder component, where the liver significantly impacts the brain via the liver-brain axis. Key mechanisms include the liver’s role in clearing peripheral β-amyloid (Aβ), the influence of hepatic enzymes and metabolites on cognitive decline, and the systemic effects of metabolic disorders on AD progression. Hepatokines, liver-secreted proteins including fibroblast growth factor (FGF)-21, selenoprotein P (SELENOP), Fetuin-A, Midbrain astrocyte-derived neurotrophic factor (MANF), apolipoprotein J (ApoJ), sex hormone-binding globulin (SHBG), Adropin and Angiopoietin-like protein 3 (ANGPTL3), could regulate insulin sensitivity, lipid metabolism, oxidative stress, immune responses, and neurotrophic support. These pathways are closely linked to core AD pathologies, including Aβ aggregation, tau hyperphosphorylation, neuroinflammation, oxidative stress and mitochondrial dysfunction. Lifestyle interventions, including exercise and dietary modifications, that regulate hepatokines expression may offer novel preventive and therapeutic strategies for AD. This review synthesizes current knowledge on the liver-brain crosstalk in AD, emphasizing the mechanistic role of liver in bridging metabolic dysfunction with neurodegeneration and underscores the diagnostic and therapeutic potential of hepatokines in addressing AD’s complex pathology.

**Supplementary Information:**

The online version contains supplementary material available at 10.1186/s13024-025-00849-6.

## Background

Alzheimer’s disease (AD) is the leading cause of dementia in older adults, responsible for about 60–70% of cases [1]. Recent research highlights AD’s systemic nature and the role of peripheral organs in its pathophysiology [2, 3]. Metabolic disorders, including insulin resistance (IR), obesity, and diabetes mellitus, have been strongly linked to an increased risk of AD, while AD itself may exacerbate these metabolic conditions, creating a pathological feedback loop. The liver plays a crucial role in metabolic homeostasis and influences nervous system function by regulating glucose, lipid, and cholesterol metabolism, as well as oxidative stress and inflammation [4, 5].

Given the central role of liver in metabolic regulation and its impact on neurophysiology, the interplay between the liver and AD pathogenesis has garnered significant research interest. Hepatokines-hormone-like proteins secreted by hepatocytes-serve as critical mediators in this context. Some hepatokines are capable of crossing the blood-brain barrier (BBB) and directly influencing nervous system function [6, 7]. Additionally, hepatokines play a role in IR and lipid metabolism in conditions like obesity, type 2 diabetes mellitus (T2DM), and other metabolic disorders, suggesting their potential as novel regulators of energy metabolism [8, 9].

Emerging evidence indicates that hepatokines, such as fibroblast growth factor (FGF)-21, selenoprotein P (SELENOP), Midbrain astrocyte-derived neurotrophic factor (MANF), Angiopoietin-like protein 3 (ANGPTL3), Fetuin-A, Adropin, ApoJ and sex hormone-binding globulin (SHBG), could regulate oxidative stress, immune responses, and apoptotic pathways, providing new insights into their broader physiological roles [10, 11]. Notably, beyond these fundamental functions, hepatokines also exhibit specific regulatory roles in AD pathology. For example, FGF-21 regulates brain insulin sensitivity, whereas ApoJ is involved in β-amyloid (Aβ) clearance, MANF is neuroprotective against tau hyperphosphorylation, and SELENOP deficiency exacerbates oxidative stress in the AD brain [12–15].

The relationship between hepatokines and AD is particularly compelling, given that metabolic dysregulation, oxidative stress, and aberrant inflammation are key drivers of AD pathology. Therefore, this review aims to investigate the mechanistic connections between AD, metabolic disorders, hepatokines, and the liver. By examining the roles of hepatokines in these pathological processes, we aim to provide novel insights into their contributions to AD and explore their potential as therapeutic targets in mitigating the progression of AD.

## The liver-brain axis and metabolic dysregulation

**Fig. 1:**
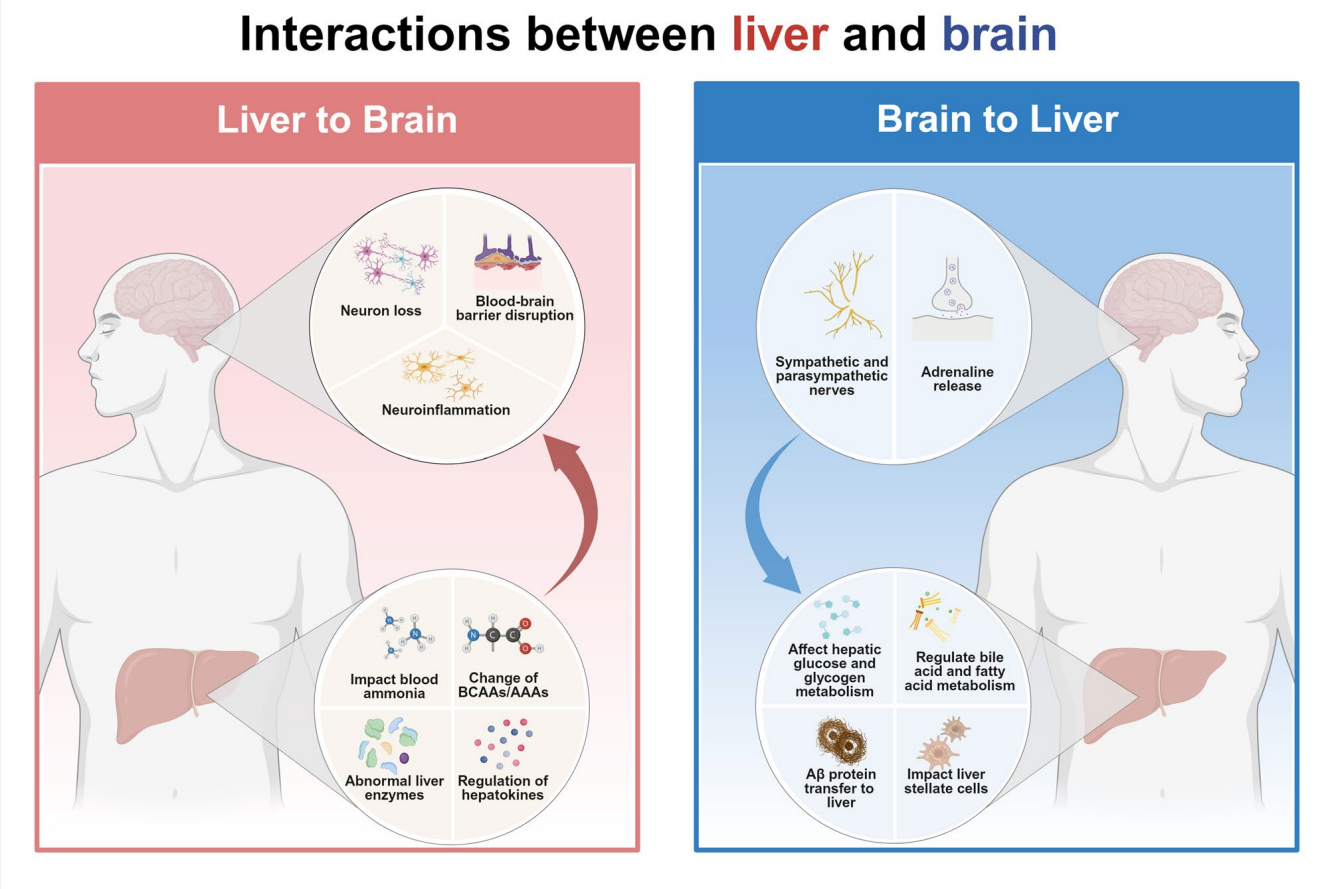
Interactions Between the Liver and Brain in Metabolic and Neurological Regulation. This figure illustrates the bidirectional communication between the liver and the CNS via metabolic and inflammatory pathways. Left Panel: The liver exerts systemic effects on the CNS through dysregulated metabolic processes and inflammatory mediators. Elevated liver enzyme activity, increased blood ammonia levels, aberrant hepatokine secretion, and an altered BCAAs to AAA ratio collectively contribute to BBB dysfunction, neuroinflammation, and neuronal degeneration. These pathological changes exacerbate CNS damage and cognitive decline. Right Panel: The brain modulates liver function through the autonomic nervous system. Sympathetic and parasympathetic innervation regulates hepatic glucose and lipid metabolism, while adrenal-mediated catecholamine release influences hepatic inflammatory responses. Furthermore, the liver plays an essential role in clearing Aβ peptides from circulation, mitigating their accumulation in the brain and reducing neurotoxicity

The liver-brain axis constitutes a bidirectional communication network between the liver and the brain mediated by the neuroendocrine system. This complex interaction regulates hepatic metabolism and modulates brain function, thereby playing a pivotal role in maintaining systemic metabolic homeostasis and contributing to the pathophysiology of various diseases (Figure.1).

### Neuromodulation of hepatic function: metabolism and inflammation

The liver is innervated by sympathetic, parasympathetic, and peptidergic nerve fibers [16], forming a crucial interface for bidirectional communication with the brain, that helps maintain metabolic homeostasis across organ systems [17]. This section explores the neuromodulation of glucose and lipid metabolism, as well as inflammation in the liver, to elucidate the role of liver-brain axis in metabolic regulation.

Elevated portal vein glucose levels trigger lateral hypothalamic activation, stimulating the parasympathetic nervous system and promoting hepatic glycogen synthesis. Conversely, when blood glucose levels decrease, the ventromedial hypothalamus activates the sympathetic nervous system, promoting hepatic glucose catabolism [18]. This mechanism ensures glucose homeostasis, with the parasympathetic system storing glucose during hyperglycemia and the sympathetic system releasing glucose during hypoglycemia. Additionally, reduced portal vein glucose levels activate the vagus nerve, prompting eating behavior to restore glucose balance [18].

The nervous system also exerts significant control over hepatic lipid metabolism. The parasympathetic activation influences hepatic bile acid metabolism, optimizing fat absorption and preventing ectopic fat accumulation. Central neuroendocrine signals, such as melanocortin and leptin, suppress lipogenesis and fat accumulation via the vagus nerve, providing an additional layer of metabolic regulation [18]. However, sympathetic activation enhances hepatic glucose uptake and stimulates glucokinase expression, indirectly supporting lipid storage and promoting lipolysis in brown adipose tissue (BAT) [19, 20]. In addition to metabolic regulation, the nervous system also directly affects liver inflammation. Sympathetic neurotransmitters such as norepinephrine and epinephrine modulate the activity of hepatic stellate cell, contributing to hepatic inflammatory reactions [21].

Overall, these interactions underscore the integral role of neural inputs in coordinating hepatic glucose and lipid metabolism, as well as inflammatory responses. These mechanisms highlight brain’s critical function in maintaining systemic metabolic equilibrium.

### Liver pathology and cognitive impairment

Cognitive impairment, a hallmark of AD, has strong links to metabolic alterations in the liver. Specifically, liver diseases such as metabolic-associated fatty liver disease (MASLD) and nonalcoholic fatty liver disease (NAFLD), have emerged as significant contributors to cognitive decline, mediated through mechanisms involving inflammation, immune dysregulation, and brain structural changes.

Patients with MASLD exhibit impairments in executive functioning and demonstrate a propensity toward global cognitive decline [22]. Moreover, in NAFLD, deficits in both immediate and delayed memory have been consistently observed, with the severity of liver fibrosis positively correlated with cognitive dysfunction [23]. Functional magnetic resonance imaging (fMRI) studies reveal that NAFLD is associated with reduced brain volume and decreased brain perfusion, indicating structural and functional compromise [24]. Weinstein et al. investigated the relationship between hepatic fibrosis and AD-related proteins using the non-invasive Fibrosis-4 (FIB-4) index. Their findings demonstrated that advanced liver fibrosis (elevated FIB-4) in NAFLD patients was associated with tau pathology in AD-vulnerable regions, including the parahippocampal gyri, inferior temporal regions and rhinal areas, while Aβ deposition was observed in specific areas such as the inferior temporal and parahippocampal regions [25, 26]. Notably, despite demonstrating a spatial correlation between the severity of hepatic fibrosis severity and AD-characteristic protein aggregation, the study did not establish a causal link between NAFLD and cognitive decline [25–27]. This difference may stem from methodological limitations inherent in the cross-sectional observation design, thus longitudinal studies are needed to elucidate temporal relationships and validate these findings.

Liver dysfunction contributes to the pathological progression of neurodegenerative diseases through multifaceted mechanisms. Metabolic dysregulation serves as the core initiating factor, while neuroinflammation is widely accepted as a central pathological process. In NAFLD or MASLD, the liver undergoes a self-perpetuating cycle of IR and lipotoxicity, leading to the dysregulation of key metabolites involved in energy homeostasis [28]. Excessive lipid accumulation within hepatocytes, along with infiltration of inflammatory immune cells, ultimately results in hepatocyte damage [26]. As part of this pathological process, hepatic lipid accumulation and immune cell infiltration trigger the sustained release of proinflammatory cytokines and chemokines, such as interleukin-6 (IL-6) and tumor necrosis factor-alpha (TNF-α), driving a systemic inflammatory response [26, 29]. Elevated circulating cytokines facilitate the recruitment of immune cells, which can penetrate the BBB and infiltrate brain parenchyma, initiating a central inflammatory cascade [26]. Chronic low-grade inflammation further induces persistent microglial activation and polarization shifts, BBB endothelial dysfunction, and mitochondrial energy metabolism abnormalities, exacerbating neuronal damage [30]. These multidimensional pathological changes collectively result in metabolic abnormalities within the liver, leading to structural remodeling of cerebral microvasculature, excessive activation of glial cells, irreversible neuronal loss, and neurotransmitter system imbalances. This cascade ultimately contributes to neurodegenerative processes.

Given the strong correlation between hepatic dysfunction, metabolic dysregulation, and neurodegeneration, recent studies have focused on identifying liver-derived biomarkers that may serve as early indicators of AD pathology. The CABLE study [31], which analyzed an AD cohort, provided compelling evidence that specific hepatic biomarkers are significantly associated with AD progression. Beyond conventional liver function markers such as aspartate transaminase (AST) and alanine aminotransferase (ALT), several other liver-derived indices exhibit characteristic changes concurrent with clinical and pathological progression of AD [31]. Specifically, total protein and globulin concentrations increase, whereas prealbumin levels and the albumin/globulin ratio decline [31]. Moreover, enzymatic activity analyses revealed that α-L-fucosidase and glutamate dehydrogenase activities are reduced by 23% and 29%, respectively, in AD patients compared to controls. Mediation analysis further demonstrated that total protein and glutamate dehydrogenase may influence cognitive function by mediating AD pathological alterations [31]. Additionally, emerging evidence from small cohort studies has identified liver-type fatty acid-binding protein 1 (FABP1) as a potential biomarker for AD pathology. Plasma FABP1 levels are significantly elevated in AD patients and are positively correlated with cerebrospinal fluid (CSF) total tau and phosphorylated tau. The plasma FABP1/Aβ_42_ or FABP1/t-tau ratios offer high discriminative value for AD diagnosis [32].

Therefore, these findings highlight the complex bidirectional interactions between liver disease and brain function. Not only do some specific proteins secreted by the liver provide diagnostic value in the early stages of AD, but diseases of the liver can also accelerate cognitive decline by inducing an immune imbalance in the brain through a systemic inflammatory response.

## The role of the liver in AD pathology

### Peripheral and hepatic clearance of Aβ

AD is marked by elevated levels of soluble and insoluble Aβ protein, along with the accumulation of neurotoxic Aβ plaques in the brain [33]. Reducing Aβ deposition through improved clearance mechanisms, alongside decreasing its production, represents a key therapeutic strategy. Research indicates that 40–60% of brain-derived Aβ protein is cleared by peripheral organs [34, 35].

Peripheral clearance of Aβ hinges on two primary mechanisms: its transport from the brain to peripheral circulation and subsequent degradation or elimination by peripheral organs. Transport across the BBB is facilitated by specific proteins, such as low-density lipoprotein receptor-related proteins 1 (LRP-1) and P-glycoprotein, which mediate the efflux of Aβ into the plasma [36, 37]. Once in circulation, Aβ is either degraded by enzymes or bound to carriers like red blood cells or lipoproteins for delivery to peripheral organs [38]. Importantly, one study found that blood Aβ markers correlated with CSF levels of Aβ_1−42_ and Aβ positron emission tomography (PET) burden in brain [39]. Measures to reduce peripheral Aβ protein levels, such as dialysis, plasma exchange, and peripheral administration of the Aβ monoclonal antibody aducanumab, also significantly reduced Aβ levels in brain [40, 41]. Besides, peripheral injection of imatinib, a γ-secretase inhibitor that does not cross the BBB, can lead to decreased plasma and brain Aβ protein levels [42, 43]. All of this evidence suggests a dynamic equilibrium between the central and peripheral Aβ pools.

Recent studies have highlighted the central role of the liver in the peripheral clearance of Aβ, along with significant roles played by the kidneys and other peripheral organs. In a parabiosis model, Tian et al. demonstrated that enhanced peripheral clearance led to a significant reduction in plasma Aβ_40_ and Aβ_42_ levels in APP/PS1 AD mice, indicating an active role of hepatic metabolism in systemic Aβ homeostasis [44]. Further supporting this, isotope tracing experiments revealed that approximately 60% of intravenously injected ^125^I labeled Aβ_1−40_ was cleared by the liver, with the remaining fraction distributed among the kidneys, gastrointestinal tract, and other peripheral organs [44]. Elevated levels of Aβ in both plasma and brain have been observed in aging and AD models, potentially resulting from impaired hepatic clearance mediated by reduced expression of liver LRP-1 receptors regulated by PPARα [45, 46]. In vitro experiments further validate this phenomenon. When fluorescently labeled synthetic Aβ_40_/Aβ_42_ was incubated with liver homogenates, the degradation rate in AD patients’ liver tissues was significantly slower than in non-demented controls [47, 48]. This difference persisted after adjusting for confounding factors such as age, sex, and apolipoprotein (Apo) E ε4 genotype [47, 48].

The impact of hepatic hemodynamic changes on Aβ metabolism further underscores the liver’s pivotal role. Experimental portal vein ligation, which reduces hepatic blood flow, was shown to simultaneously increase Aβ levels in blood and CSF, revealing a dynamic balance between hepatic clearance efficiency and cerebral Aβ burden [46]. This balance is particularly fragile in patients with chronic liver disease. Clinical observations have shown significantly elevated plasma Aβ_40_/Aβ_42_ concentrations in patients with liver dysfunction, such as cirrhosis or hepatitis B-related liver impairment [49]. These findings suggest that liver disease may exacerbate AD pathology through two mechanisms: diminished peripheral clearance capacity and increased production of pathogenic proteins due to liver dysfunction.

As research progresses, the liver has been found to be not only a clearing organ but also a potential systemic source of Aβ. The hepatocyte-specific human amyloid transgenic mouse model developed by Lam et al. demonstrated that hepatic expression of human amyloid precursor protein significantly increased Aβ levels in both plasma and brain tissues [50]. Mechanistic studies revealed that liver-synthesized Aβ is transported across the BBB via apolipoprotein B (ApoB)-dependent lipoprotein complexes [50]. This aberrant transport pathway was associated with accelerated age-related amyloid plaque deposition observed in PET imaging and progressive hippocampal and cortical volume atrophy evident in MRI scans [50].

Genomic analyses further highlight the liver’s involvement in AD pathology. Significant expression of AD-associated genes in liver tissues points to a potential genetic link between hepatic function and Aβ metabolism [51]. This genetic evidence, coupled with the liver’s central role in systemic Aβ clearance, emphasizes its critical function in reducing brain Aβ burden and suggests therapeutic opportunities targeting hepatic Aβ clearance pathways.

In addition to the liver, other peripheral organs can contribute to Aβ elimination [52]. Studies have shown that unilateral nephrectomy in APP/PS1 mice exacerbates brain Aβ deposition and aberrant phosphorylation of tau proteins [53]. The spleen, as the largest peripheral immune organ, participates in Aβ clearance through monocyte/macrophage-mediated phagocytosis. Experimental splenectomy in AD mice resulted in a twofold increase in circulating Aβ_40_ levels, along with an enlargement of cortical amyloid plaques [52].

### Peripheral and hepatic clearance of Tau protein and α-synuclein

Tau protein and α-synuclein (α-syn) aggregation are two additional neuropathological hallmarks of neurodegenerative disorders, including AD and Parkinson’s disease (PD) [54, 55]. Recent studies indicate that abnormal deposits of tau and α-syn are not confined to the central nervous system (CNS) but can also be detected in peripheral organs, including the liver.

Physiological clearance of tau protein involves a dynamic equilibrium between the CSF and systemic circulation [55]. Experimental studies have demonstrated that following the intracisternal injection of exogenous tau in wild-type mice, plasma tau levels rise significantly within minutes, suggesting rapid systemic distribution [55]. Concurrently, peritoneal dialysis effectively reduces tau concentrations in the brain’s interstitial fluid, reinforcing the hypothesis that peripheral clearance mechanisms may influence central tau pathology [56]. Notably, parabiosis models further indicate that systemic factors and peripheral organs may exert regulatory effects on tau accumulation in the brain [55]. However, the specific molecular mechanisms underlying the tau clearance via the liver-brain axis remain to be elucidated. Similar to tau, α-syn exhibits a bidirectional transport dynamic across the BBB [57]. Radiolabeling experiments have confirmed that α-syn can efficiently cross the BBB, facilitating its systemic dissemination via the circulatory system [58]. Remarkably, pathological α-syn deposition has been detected in the liver one month after a single striatal injection in murine models, suggesting a brain-to-periphery transmission route. Furthermore, in transgenic mouse models expressing human α-syn, hepatic α-syn protein levels exhibit time-dependent accumulation despite the absence of α-syn mRNA expression in the liver under neuron-specific promoter regulation, suggesting a potential neural system-derived origin of the protein [57]. Additionally, the vagus nerve and visceral nerve branches form direct neural connections with the liver in the portal tract, providing a possible inter-organ transport route for α-syn [59].

### Liver function markers in AD-related cognitive impairment

In addition to Aβ clearance, the liver is crucial for metabolic regulation, detoxification, protein synthesis, and immune support, and its dysfunction has been implicated in AD [2]. Studies indicate that liver metabolic changes may precede cognitive decline in AD, and that hepatic metabolic dysfunction can also occur in advanced AD [2].

Elevated liver enzymes, indicative of hepatic dysfunction, are non-linearly associated with cognitive impairment in AD [60]. Elevated liver enzymes correlate with higher plasma Aβ levels, suggesting that maintaining normal enzyme levels may help lower cognitive impairment risk [61]. It is noteworthy that even fluctuations in ALT levels within the upper limits of the normal reference range may indicate subclinical hepatic dysfunction [48]. The association between liver function biomarkers and AD pathology remains inconsistent across studies. A ten-year longitudinal cohort study demonstrated that increased ALT levels were positively correlated with the rate of cerebral Aβ deposition [48]. However, a cross‐sectional analysis of the ADNI cohort yielded contrasting results, showing that serum ALT levels in AD patients were significantly lower compared to cognitively normal controls. Moreover, these reduced ALT levels were associated with greater amyloid PET burden and accelerated cognitive decline [62]. The observed discrepancies stem from differences in study design and methodology. The longitudinal study accounted for dynamic changes in hepatic function over time, whereas the cross-sectional ADNI study lacked prospective data support. Additionally, confounding factors such as alcohol consumption, metabolic syndrome, and systemic inflammation were not consistently controlled across studies, potentially influencing the reported associations [62]. Moreover, most current evidence indicates that AST levels are not significantly associated with cognitive impairment [62]. However, some experiments have shown that a decreased ALT/AST ratio is not only linked to the accumulation of Aβ protein and cerebral atrophy but also related to impaired glucose metabolism in the frontal, parietal, and temporal lobes [2]. In addition, gamma-glutamyl transferase (GGT) levels exhibit a negative association with cognitive performance, while elevated alkaline phosphatase (ALP) levels increase the risk of Aβ deposition, PET burden, and cognitive impairment [60].

Genetic factors, particularly the ApoE ε4 allele, the strongest genetic risk factor for sporadic AD, have been implicated in modulating these relationships. In ApoE ε4 carriers, increased AST/ALT ratios and reduced ALT levels were significantly associated with higher brain Aβ load and accelerated cognitive decline [63]. This association, however, appears to be absent or attenuated in non-ApoE ε4 carriers, suggesting a genotype-dependent interaction between hepatic dysfunction and neurodegenerative processes.

Overall, while the exact role of liver enzymes in AD remains to be fully elucidated, these findings indicate that the observed associations might be mediated by hepatic alterations linked to ApoE4. Besides, structural brain changes in cortical and subcortical regions, including the thalamus and hippocampus, are also significant alterations in ALT, GGT, and AST/ALT ratios [64]. Furthermore, age and sex influence the link between liver function and cognitive impairment, with older adults and women showing heightened susceptibility to cognitive decline in the context of lower liver enzyme levels [65].

In addition to enzymes, liver-derived metabolites such as branched-chain amino acids (BCAAs) and ammonia are critically involved in AD-related cognitive dysfunction. Altered blood BCAAs levels and ammonia reflect liver dysfunction linked to enzyme imbalances [66]. In hepatic encephalopathy, elevated blood ammonia resulting from liver failure disrupts the BBB via the Nuclear Factor Κ-Light-Chain-Enhancer of Activated B Cells (NF-κB) pathway, impairs mitochondrial function in astrocytes, and perturbs neurotransmitter balance through potassium dysregulation [67]. Ammonia can further inhibit long-term potentiation in the hippocampus, thereby impairing memory and learning [68]. In addition, hepatic dysfunction leads to imbalances in amino acids, with reductions in BCAAs and increases in aromatic amino acids (AAA). This results in a lower ratio of BCAAs to AAA, which may disrupt neurotransmitter synthesis and exacerbate neurological dysfunction [69]. In addition, human serum albumin (HSA) is synthesized by the liver, and albumin levels decrease in hepatic impairment. HSA acts as an endogenous inhibitor of Aβ and prevents Aβ deposition associated with AD [70]. Multidimensional techniques such as time-resolved fluorescence and fluorescence correlation spectroscopy (FCS) quantitatively revealed that HSA has differential binding and modulation of Aβ monomers and aggregates [71, 72]. The binding constant of HSA to Aβ_42_ was measured to be significantly higher than that of Aβ_40_, an affinity gradient that corresponds to the greater pathogenicity of Aβ_42_, suggesting that HSA has a selective advantage in targeting the more neurotoxic Aβ isoforms [71]. Molecular dynamics simulation techniques also revealed that HSA forms a multimodal binding to the Aβ_42_ monomer, preferentially anchors to HSA structural domain III, and remodels the conformational distribution of Aβ_42_ through promiscuous interactions [72]. Therefore, when liver function is impaired, the reduction of HSA also decreases Aβ clearance efficiency and accelerates pathological changes in AD.

While markers of liver function, such as liver enzymes, are increasingly recognized as biomarkers for AD risk, the causal relationships between hepatic dysfunction and cognitive pathology remain unclear. Nonetheless, this link further confirms the interaction between the liver and brain in AD.

### The crosstalk between the liver, metabolic disorders, and AD

As a central organ in the regulation of human metabolism, the liver plays an indispensable role in maintaining the balance of glucose and lipid metabolism [73]. However, the development of metabolic disorders, such as dyslipidemia, IR, and hyperglycemia, not only impairs the normal function of the liver, but also triggers a series of complex pathological processes [73]. For example, in NAFLD, excessive lipid uptake by the liver works in conjunction with impaired clearance, resulting in an abnormal lipid accumulation [74]. This leads to the subsequent triggering of an inflammatory response and apoptosis, which accelerate the progression of steatohepatitis. As the pathological burden increases, this metabolic dysregulation may eventually evolve into hepatic fibrosis, cirrhosis, or even further development of hepatocellular carcinoma [75].

It is also important to note that hepatic dysfunction is not limited to the liver itself. Its pathological effects can have a cascading impact on metabolic regulatory networks, affecting multiple organs, including the CNS [76]. A strong association has been demonstrated between metabolic disorders and the onset and progression of AD in studies [77]. IR has been demonstrated to significantly impair insulin signaling in neurons, which in turn affects synaptic plasticity and memory function [78]. Additionally, abnormalities in lipid metabolism, particularly elevated triglyceride (TG) to high-density lipoprotein (HDL) cholesterol ratios, have been identified as an important risk factor for rapid cognitive decline and an increased pathologic burden of disease [77]. HDL functionality, rather than absolute HDL levels, may be the key determinant in AD pathophysiology. Specifically, HDL function is tightly linked to its primary protein component, apolipoprotein A-I (ApoA-I), which plays a critical role in lipid metabolism [79]. Recent studies have reported a significant reduction in circulating ApoA-I levels in AD patients compared to cognitively healthy individuals, suggesting that ApoA-I deficiency, rather than HDL quantity alone, may contribute to increased AD risk [80].

Metabolic disorders serve to further exacerbate the pathological progression of AD through a number of different mechanisms [76]. For instance, vascular risk factors, such as hypertension and reduced low-density lipoprotein (LDL) levels, result in disruption of the BBB structure, which accelerates the accumulation of Aβ deposition and tau pathology [77]. Concurrently, chronic metabolic disorders stimulate microglia and astrocytes, leading to a sustained inflammatory response. This inflammatory environment directly contributes to the deterioration of neurodegenerative pathologies [81].

The liver is the main site of ApoE synthesis and secretion, and the ApoE gene, especially the ε4 allele, is the strongest genetic risk factor for sporadic AD, and some of the AD disease markers and therapeutic effects are even dependent on differences in ApoE genotype [82, 83]. Lipidomic analysis of hepatocytes with different ApoE genotypes revealed that ApoE ε4 may promote systemic metabolic imbalance by interfering with lipid distribution, glucose metabolism, and adipose tissue inflammation [84].

The ApoE ε2/ε3/ε4 polymorphism represents a significant genetic determinant of individual variations in lipid and lipoprotein levels [85]. Compared to the ε3 allele, the ε2 allele is typically associated with lower plasma cholesterol and LDL cholesterol levels but higher TG levels, whereas the ε4 allele is linked to elevated levels of total cholesterol, LDL cholesterol, and TG [86]. This genotype-phenotype association is modulated by environmental factors such as sex, nutritional status, and metabolic disorders. Furthermore, ApoE enhances the interaction of lipoproteins with hepatocyte surface proteoglycans, thereby facilitating the hepatic uptake of ApoB-containing particles [87].

Although the traditional view holds that the BBB separates peripheral and CNS sources of ApoE, emerging evidence indicates that peripheral ApoE exerts significant regulatory effects on CNS function [86]. To address this scientific controversy, researchers have developed transgenic mouse models that specifically express human ApoE3 or ApoE4 in the liver. These models allow for a systematic investigation of the differential effects of peripheral ApoE isoforms on brain function and AD pathology [85]. By employing a conditional gene expression strategy on an ApoE knockout background to eliminate interference from endogenous central ApoE, the study demonstrated that peripheral expression of ApoE4 alone is sufficient to induce memory impairment, BBB leakage, and perivascular gliosis, with a severity comparable to that observed in systemic ApoE4 expression models [88]. It is noteworthy that a study using a humanized liver ApoE ε4/ε4 mouse model found that mice with the ApoE ε4/ε4 genotype exhibited significantly lower plasma ApoE4 levels compared to those with the ApoE ε2/ε3 genotype. This reduction was not only accompanied by downregulation of endogenous mouse ApoE expression in the brain, but also decreased the levels of synaptic glutamate receptors, presynaptic protein α-syn [89]. In addition, single-cell transcriptomic analysis of a mouse model of hepatic ApoE3 knockout revealed that hepatic ApoE3 significantly upregulates genes associated with cell adhesion, synaptic plasticity, and carbohydrate metabolism [88, 90]. In contrast, ApoE4 drives dysregulation of lipid metabolism, endothelial inflammation, and abnormal activation of extracellular matrix remodeling pathways, which directly leads to widespread downregulation of astrocytic end foot associated genes in the BBB [88, 91]. Proteomic studies further confirmed that ApoE3 improves cerebrovascular function by inducing extracellular matrix-stabilizing proteins such as Timp3, whereas ApoE4 exacerbates pathological progression by suppressing lipoprotein metabolism-related proteins and activating the complement system [88, 91].

In conclusion, the liver plays a pivotal role in the metabolic regulatory network, and its dysfunction not only affects the course of liver disease but is also closely related to the development of neurodegenerative diseases. Uncovering the interactions between liver dysfunction, metabolic disorders, and AD will provide a novel perspective and theoretical basis for understanding disease mechanisms and developing intervention strategies.

### Emerging role of hepatokines in AD

**Fig. 2:**
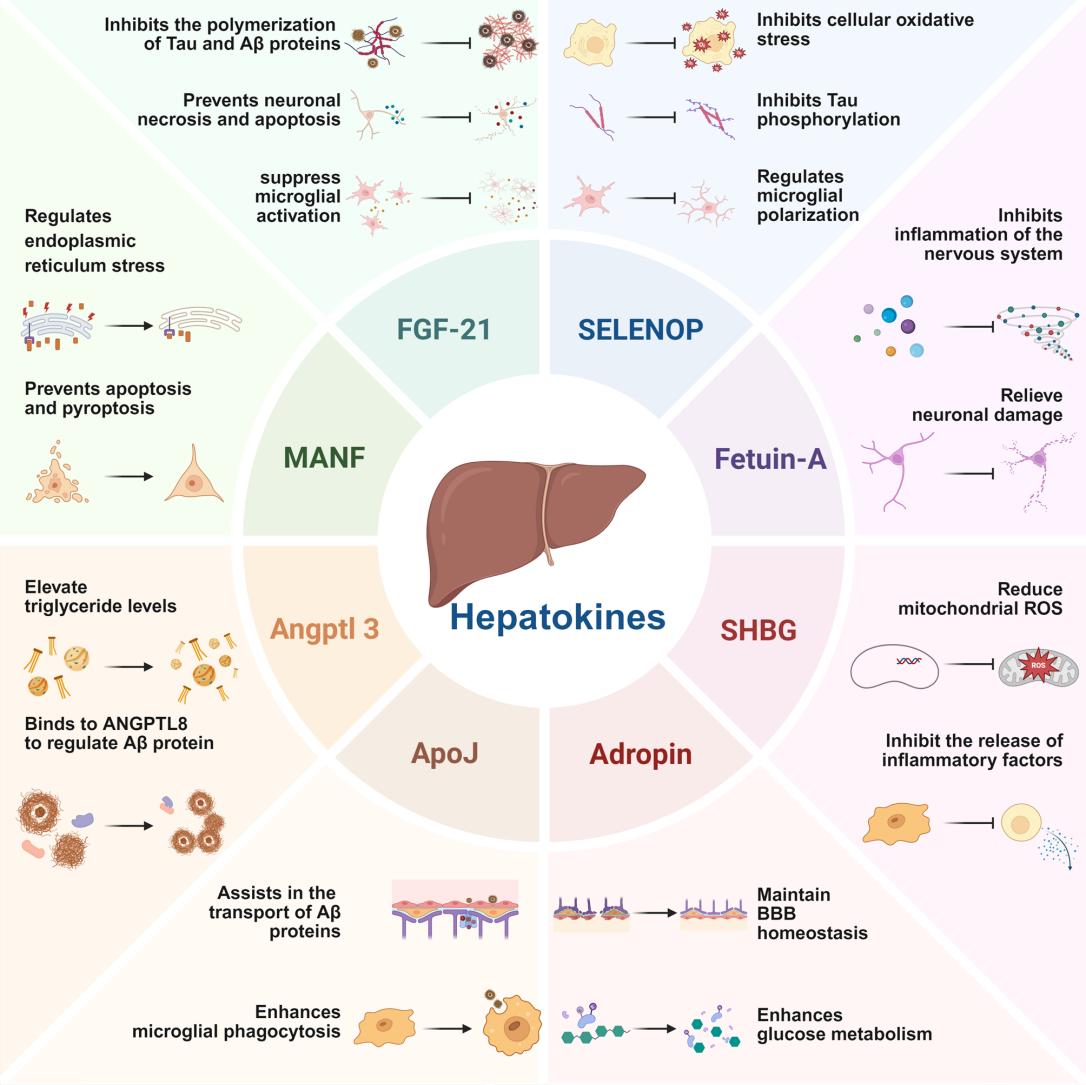
Roles of Hepatokines in Alzheimer’s Disease. This figure summarizes the multifaceted roles of hepatokines in the pathophysiology of AD, highlighting their contributions to inflammation, oxidative stress, and neuronal function. FGF-21 inhibits tau and Aβ polymerization, regulates microglial polarization to suppress inflammation, and protects neurons from necrosis and apoptosis. SELENOP delivers selenium to the brain, mitigating oxidative stress, inhibiting tau phosphorylation, and suppressing M1 microglial activation to regulate neuroinflammation. Fetuin-A reduces mitochondrial oxidative stress, prevents neuronal apoptosis, and inhibits the release of pro-inflammatory factors. By modulating testosterone, SHBG enhances mitochondrial function, which in turn reduces oxidative stress and inflammation. Adropin strengthens the BBB, facilitates glucose metabolism, and reduces oxidative damage. ApoJ aids in Aβ clearance, boosts microglial phagocytosis, and alleviates neuroinflammation. ANGPTL3, interacting with ANGPTL8, regulates lipid metabolism and contributes to neuroinflammation and Aβ dysregulation. Lastly, MANF alleviates ER stress, suppresses oxidative damage, and promotes anti-inflammatory microglial activity, underscoring its neuroprotective potential

Hepatokines, active molecules secreted by the liver that function like hormones and regulate glucose and lipid metabolism, thereby maintaining whole-body homeostasis. In addition to peripheral effects, certain hepatokines, such as FGF-21, SELENOP, Fetuin-A, ANGPTL3, MANF, Adropin, ApoJ, and SHBG, exhibit direct or indirect effects on the CNS and represent promising therapeutic targets for AD (Figure.2).

### FGF-21

FGF-21, a member of the fibroblast growth factor (FGF) family, is produced in the liver, pancreas, gastrointestinal tract, and adipose tissue. Notably, FGF-21 can cross the BBB, directly impacting neuronal and glial function [92].

Based on studies involving APP/PS1 transgenic mice, FGF-21 has been shown to reduce Aβ plaque deposition and suppress pathological tau hyperphosphorylation in AD model mice by modulating monocarboxylate transporter-dependent metabolic pathways [93]. Besides, FGF-21 could promote brain-derived neurotrophic factor (BDNF) production, reducing hippocampal neuronal death and autophagy [94]. It also activates key signaling cascades, including PI3K/AKT/GSK-3β and PP2A/MAPKs/HIF-1α, to counteract oxidative stress and neuronal death [93, 95]. FGF-21 also modulates neurotransmitter metabolism, influencing aspartate and GABA levels to reduce neuronal damage [96, 97]. Anti-inflammatory effects of FGF-21 further contribute to its neuroprotection. It inhibits M1 microglial activation and downregulates pro-inflammatory cytokines release through NF-κB signaling, thereby mitigating neuroinflammation [93, 98].

Multiple-timepoint regression analysis was employed to quantify the blood-to-brain influx of FGF-21 across the BBB. Intravenous administration revealed distinct pharmacokinetic characteristics: serum analysis demonstrated 94% molecular integrity of FGF-21 at 10 min post-infusion, while intracerebral measurements showed 75% preservation of intact molecules. The influx rate was 0.23 ± 0.12 µl/g-min, almost four times faster than the vascular marker albumin. At 10 min, approximately 0.5% of administered FGF-21 was present per gram of brain tissue, with 70% reaching the brain parenchyma [6]. ‌Therefore, FGF-21 may cross the impaired BBB in AD. Additionally, there have been studies utilizing materials science to improve the therapeutic effect of FGF-21 for AD. Wang et al. has developed PEGylated liposomes modified with BV2 microglial cell membranes (hFGF-21@BCM-LIP), enabling targeted delivery of liver-derived FGF-21 to AD pathological microenvironments via subcutaneous injection [99]. Post-treatment analysis revealed that FGF-21 reduced BACE1 levels in the brain and reversed the inflammatory imbalance between IL-6, TNF-α and IL-10, thereby significantly improving the cognitive function of AD models [99]. Besides, Rühlmann et al. performed in vitro experiments using hippocampal organotypic brain slice cultures and demonstrated that the FGF-21 analog LY2405319 upregulates the mRNA expression levels of PPARγ, ApoE, and ABCA1, while also reducing the number and size of Aβ plaques [100]. In AD animal models, FGF-21 analog significantly improved neuronal structural integrity and inhibited excessive microglial activation. Although the analog failed to reduce Aβ plaques burden in late-stage AD models, it ameliorated neuroinflammation and energy metabolism imbalance [100]. Additionally, the co-administration of FGF-21 with rapamycin amplifies these effects by promoting Aβ clearance, reducing autophagy-related protein accumulation, and increasing neuronal density [101].

FGF-21 can also play a regulatory role in other neurodegenerative diseases. Mitochondrial dysfunction in PD activates the integrated stress response regulator activating transcription factor 4 (ATF4), triggering neuroprotective mechanisms. Under pathological conditions, mitochondrial damage not only promotes translational activation of ATF4, but also induces transcriptional upregulation of the ROS-p38 MAPK-activating transcription factor 2 (ATF2) signaling axis driven by FGF-21 [102]. However, excessive activation of ATF4 in PD models is pathologically associated with progressive loss of dopaminergic neurons in the substantia nigra. FGF-21 may regulate mitochondrial damage and oxidative stress responses by balancing the protective and toxic effects of integrated stress response activation [103]. Besides, in PD mice, FGF-21 suppresses neuroinflammation and apoptosis, enhancing neuronal resilience to metabolic stress and ultimately preserving the functional integrity of the dopaminergic system [104].

### SELENOP

Selenium (Se) is an essential trace element critical for human health, with approximately 2.3% of the body’s total selenium residing in the brain [105]. Despite being present in trace amounts, both deficiency and excess of Se can significantly affect the nervous system by influencing neuronal activity. Selenium is incorporated into selenoproteins as selenocysteine (Sec), playing a pivotal role in Se transport and regulation [105].

Among the primary human selenoproteins, SELENOP and glutathione peroxidase 3 (Gpx3) play crucial roles in selenium metabolism. While Gpx3 is predominantly synthesized in the kidneys, SELENOP is a heparin-binding glycoprotein produced and secreted by the liver. SELENOP accounts for 53% of plasma selenium, compared to Gpx3’s 19%, highlighting its central role in maintaining systemic Se homeostasis [106]. The mRNA structure of SELENOP includes up to 10 s residues, with nine located at the C-terminal end responsible for Se transport, and one at the N-terminal end serving as the active site of enzyme [106]. The mRNA of SELENOP features a UXXC sequence, similar to the CXXC sequence in thioredoxin, but sulfur is replaced by Se, providing it the capacity to resist oxidative stress [106].

Selenium demonstrates substantial neuroprotective properties. It can diminish oxidative stress, prevent DNA damage, and improve mitochondrial function and biogenesis [107]. As the principal selenium transporter in the brain, SELENOP crosses the BBB via endocytosis by brain endothelial cells [108]. Once in the CNS, SELENOP mitigates oxidative damage by eliminating excess free radicals and bolsters neural function by modulating the cellular antioxidant defense system. In addition, SELENOP exerts anti-inflammatory effects by inhibiting the polarization of microglia towards the pro-inflammatory M1 phenotype and reducing the release of pro-inflammatory cytokines [109, 110].

With the progress of research, the potential role of SELENOP in AD has gradually emerged. SELENOP is highly expressed in the hippocampus and cortex, key regions affected in AD pathology [111]. SELENOP reduces tau aggregation by modulating tau protein phosphorylation and promotes the disaggregation of pre-formed tau tangles [111]. Besides, selenium nanoparticles, composed of nanoscale selenium particles, have been shown to reduce Aβ aggregation [112]. During the progression of AD, impaired compartmentalization of copper and zinc ions is one of the factors that trigger neuronal degeneration. As a regulator of metal homeostasis, SELENOP functions through the ApoE receptor 2 encoded by the ApoE gene [113]. Notably, polymorphisms in the ApoE gene have been shown to be strongly associated with genetic susceptibility to AD, and zinc ions specifically cleave ApoE proteins to produce shorter fragments. In contrast, binding of SELENOP to zinc ions helps to maintain the stability of ApoE length, thereby inhibiting Aβ aggregation [113, 114]. Two studies using recombinant expression of the histidine-rich domain of SELENOP (SelP-H) in neuroblastoma cells revealed the molecular mechanism by which SELENOP intervenes in Aβ aggregation and neurotoxicity through metal ion homeostasis. Isothermal titration calorimetry confirmed that the binding affinities of SelP-H for Cu⁺ and Cu²⁺ were sub-picomolar and nanomolar, respectively, while those for Zn²⁺ and Cd²⁺ were in the micromolar range [115, 116]. Transmission electron microscopy showed that by chelating metal ions, SelP-H inhibited Aβ fibrillation and significantly reduced the abnormal aggregation tendency of Aβ_42_ [115, 116]. In addition, cellular experiments showed that SelP-H not only reduced intracellular ROS levels, but also reduced neurotoxicity induced by Cu⁺/Cu²⁺-Aβ_42_ or Zn⁺-Aβ_42_ complexes by 40–60% [115, 116]. These findings suggested that SELENOP may provide neuroprotection through dual mechanisms of metal chelation and antioxidant activity [115, 116]. However, it has also been suggested that the interaction of SELENOP with Aβ and neurofibrillary tangles may exacerbate oxidative stress in AD by impeding Se delivery to neurons and glial cells [117].

Furthermore, the heightened oxidative stress in AD increases Se demand in nerve cells, which contributes to the increased levels of SELENOP. Intriguingly, the relationship between SELENOP levels in plasma and CSF and cognitive impairment appears nonlinear. Both abnormally low and high SELENOP levels are associated with adverse effects on nervous system function, suggesting a delicate balance in Se regulation is critical for maintaining neural health [118, 119].

Crucially, the transport efficiency of SELENOP across the BBB directly determines its bioavailability in neurons. An in vitro transwell BBB model demonstrated that exosomes secreted by HepG2 cells could carry SELENOP and penetrate a 0.4 μm pore-size membrane structure composed of bEnd.3 endothelial cells and N2a neurons, reaching the neuronal compartment via a non-ApoE-dependent pathway [112]. This led to a dose-dependent increase in SELENOP levels in N2a cells [112].

SELENOP may also play a regulatory role in other neurodegenerative diseases. In PD, CSF selenium levels are significantly correlated with SELENOP concentrations. Immunohistochemistry reveals that SELENOP is specifically enriched in the core regions of Lewy bodies within the substantia nigra pars compacta and co-localizes with α-syn, suggesting its potential role in regulating abnormal protein aggregation [120]. Although SELENOP expression in living dopaminergic neurons in PD patients is compensated and upregulated, the area of SELENOP regulation in the substantia nigra is relatively small, so it may not be directly used as a valid biomarker for PD [121]. These findings indicate that SELENOP could influence the progression of neurodegenerative pathology, warranting further investigation.

### Fetuin-A

Fetuin-A, a negatively charged glycoprotein weighing around 63 kDa, is primarily synthesized in liver parenchymal cells, with minor contributions from macrophages and adipocytes [122]. It is widely distributed in brain tissue, the choroid plexus, the gastrointestinal system, and kidneys. As a multifunctional plasma carrier protein, Fetuin-A is pivotal in various pathological conditions, including NAFLD, T2DM, cardiovascular, and neurodegenerative diseases [123].

Neuroinflammation is a critical factor in the onset and progression of AD. Chronic inflammation in the brain triggers immune activation and the release of pro-inflammatory agents, impairing the clearance of neurotoxic substances or directly harming neurons [81]. Fetuin-A exerts potent anti-inflammatory effects in the CNS. Studies have demonstrated its ability to reduce plasma levels of pro-inflammatory cytokines such as IL-6 and TNF-α [124]. ​​Mechanistically, Fetuin-A inhibits the activation of high-mobility group box 1 (HMGB1), a key mediator in AD pathogenesis. This inhibition underlies its anti-inflammatory effects [125]. Furthermore, due to its structural homology with the type II TGF-β receptor, Fetuin-A suppresses the TGF-β signaling pathway—a critical regulator of immune responses and neuronal repair [126]. It also mitigates mitochondrial oxidative stress in microglia exposed to glutamate and reduces excitotoxicity-induced neuronal apoptosis via activation of the Nrf-2/HO-1 pathway [127]. However, in the context of neuroinflammatory conditions such as AD, reduced Fetuin-A levels have been reported. Clinical evidence indicates that levels of plasma Fetuin-A positively correlate with Mini-Mental State Examination (MMSE) scores [128]. Moreover, in AD patients, these levels are significantly lower compared to age-matched healthy controls [128].​​ This reduction may stem from compromised BBB integrity, which impairs the transmembrane transport of endogenous Fetuin-A [7]. ​​Notably, Ricken et al. extended this finding by revealing that phosphorylation modifications of liver-derived Fetuin-A in human serum and CSF are linked to CNS inflammation in AD, suggesting that post-translational regulation may further impair its functionality under pathological conditions [7].

​​Importantly, the absolute concentration of phosphorylated Fetuin-A in CSF (0.07 ± 0.16 µg/ml) was markedly lower than in serum (27 ± 2 μg/ml), and it was detected in approximately half of the CSF samples [7]. Moreover, the CSF/serum ratio of phosphorylated Fetuin-A showed a significant positive correlation with the BBB permeability index, as measured by the CSF/serum albumin ratio [7]. This finding suggests that peripheral Fetuin-A may traverse a leaky BBB to exert central effects. Of therapeutic significance, exogenous Fetuin-A supplementation demonstrates potential as a clinical intervention [126]. By crossing the BBB in a dose-dependent manner, exogenous Fetuin-A mitigates neuronal damage and may compensate for deficiencies arising from both impaired transport and dysfunctional post-translational modifications [126].

Recent studies have demonstrated differential expression patterns of Fetuin-A in neurological disorders such as PD, amyotrophic lateral sclerosis (ALS), and multiple sclerosis (MS) [129–131]. In PD, multiple investigations using mouse models and postmortem patient analyses have consistently shown significantly reduced levels of fetuin-A, with its expression being crucial for maintaining the density and functionality of Purkinje cell dendritic spines [129]. In contrast, studies on ALS reveal that proteomic analyses detect significantly elevated CSF levels of the fetuin-A precursor in rapidly progressing cases compared to those with slower disease progression [130]. Furthermore, research on MS has shown that elevated CSF Fetuin-A levels are associated with active disease states. These levels decrease following natalizumab treatment, with the reduction inversely correlating with improvements in Expanded Disability Status Scale (EDSS) scores [131]. Overall, these findings not only highlight the unique expression patterns of Fetuin-A across different neurological conditions but also underscore its potential role in disease pathogenesis and progression.

### ANGPTL3

ANGPTL3 is exclusively synthesized in the liver and secreted into circulation, thus recognized as a liver-regulated metabolic factor [132]. ANGPTL3 exerts significant inhibitory effects on key lipases in lipid metabolism. Its N-terminal domain inhibits lipoprotein lipase (LPL), raising plasma TG levels. It also inhibits endothelial lipase, reducing HDL catabolism and thereby increasing HDL-C levels [133]. The modulation of ANGPTL3 activity significantly impacts lipid profiles. Functional inactivation of ANGPTL3 decreases plasma levels of TG, very low-density lipoprotein (VLDL), and low-density lipoprotein cholesterol (LDL-C) [134], while overexpression of ANGPTL3 or exogenous supplementation with its protein increases TG levels [135].

Lipid metabolism dysregulation is intricately linked to AD pathogenesis. Elevated levels of LDL are known to trigger oxidative stress and neuroinflammation, exacerbating AD progression, whereas HDL exerts protective effects by facilitating Aβ clearance and suppressing inflammation [136]. Since ANGPTL3 elevates LDL levels, it may indirectly exacerbate neuroinflammation in AD. Furthermore, ANGPTL3 forms heterodimers with ANGPTL8, which synergistically influence processes such as lipid metabolism and inflammation [137].

ANGPTL3 is primarily recognized for its role in regulating lipid metabolism. Studies in mice have demonstrated that its overexpression leads to increased plasma lipid levels. In degenerative diseases such as MS, lipid metabolism is critical due to the demyelination process, which involves damage to the lipid-rich myelin. Effective clearance of myelin debris is essential for remyelination and repair, indicating that ANGPTL3 may hold potential therapeutic value [138].

### MANF

MANF, a protein situated in the endoplasmic reticulum (ER), plays a key role in neuronal development and apoptosis regulation [139]. The ER maintains cellular homeostasis through processes like ER-associated degradation and the unfolded protein response (UPR), which manages protein folding during stress [140, 141]. However, prolonged stress can overwhelm these processes, disrupting ER homeostasis and triggering apoptosis via the C/EBP homologous protein pathway [142].

MANF is predominantly situated on the luminal side of the ER, featuring a C-terminal RTDL sequence that interacts with the KDEL receptor to retain ER and Golgi membrane proteins [143]. Under unstressed conditions, MANF and glucose-regulated protein 78 (GRP78) are expressed at low levels. As stress levels increase, however, GRP78 competitively binds to KDEL receptors, resulting in an increase in free MANF, the dynamic competition between GRP78 and MANF leads to the eventual secretion of MANF [144, 145]. Therefore, the extent of MANF secretion varies with stress intensity, with higher stress levels leading to more extracellular release. MANF plays a dual role in relieving ER stress and promoting neuronal survival. It mitigates UPR signaling by reducing XBP1 mRNA levels and inhibiting Ire1α oligomerization and phosphorylation [142]. Additionally, MANF reduces oxidative stress by decreasing reactive oxygen species (ROS) levels and promotes neuronal differentiation via the PI3K/AKT/mTOR pathway [142].

Research has demonstrated that MANF is elevated in 8-month-old APP/PS1 mice and colocalizes with the ER stress markers BiP and CHOP [146]. MANF reduces the expression of the pro-apoptotic factor CHOP by inhibiting the nuclear translocation of activating transcription factor 6 (ATF6), downregulating the generation of the XBP1s splice variant, and blocking activation of the PERK-eIF2α pathway [146]. Notably, overexpression of MANF or treatment with recombinant human MANF could directly reduce Aβ-induced neuronal apoptosis and ER stress [146]. Furthermore, MANF modulates microglial polarization via the A20/NF-κB pathway, fostering an anti-inflammatory state and safeguarding neurons against apoptosis [147].

Although MANF is predominantly a liver-secreted, feeding-induced hepatic factor, it is also secreted by neurons and glial cells in the nervous system [148]. Under pathological conditions, endogenous MANF secretion may be insufficient to counteract severe ER stress. Peripheral supplementation of MANF has shown potential to reduce infarct volume and neuroinflammation in ischemic stroke models, highlighting its therapeutic promise for other neurological diseases [149].

Recent studies have demonstrated that MANF exhibits neuroprotective effects against various neurodegenerative diseases. In an experimental autoimmune encephalomyelitis model, dexamethasone treatment significantly increased MANF expression in the myelin regions of spinal cord white matter, highlighting its potential as a therapeutic target for MS [150]. Notably, the protective mechanism of MANF in the dopaminergic system has been systematically validated in PD models. Early studies in Drosophila revealed that MANF deficiency leads to axonal degeneration in dopaminergic neurons, while exogenous supplementation with MANF rescued neuronal survival [151]. This mechanism was further supported in C. elegans models, where MANF knockout resulted in the loss of approximately 33% of dopaminergic neurons. In α-syn toxicity models, MANF overexpression significantly delayed neurodegeneration by correcting dopaminergic metabolic disturbances [151]. In mammalian models, MANF has been shown to reverse MPTP-induced reductions in striatal dopamine and its metabolites DOPAC and HVA, thereby improving motor function. The multi-targeted actions of MANF underscore its potential as a candidate molecule in the treatment of neurodegenerative diseases [151].

### Adropin

Adropin, a conserved 76-amino acid peptide, is primarily synthesized in the liver and functions as a hormone-like factor involved in energy homeostasis, glucose regulation, and lipid metabolism [152, 153]. While its expression is most prominent in the liver, Adropin is also present in the brain, pancreas, and plasma [154]. Its physiological effects are mediated through receptors such as Gpr19 and Nb3/Contactin6 [155]. Binding to Gpr19 activates PI3K/AKT signaling pathway, leading to AKT phosphorylation at Ser^473^ [156]. This phosphorylated AKT plays a central role in regulating cell proliferation, differentiation, angiogenesis, neuronal regeneration and synaptic plasticity through mTOR signaling [155].

In AD, BBB disruption exacerbates neuroinflammation and oxidative stress, further aggravating neuronal damage. BBB disruption also enhances β- and γ-secretase activity, promoting increased Aβ production [157]. In maintaining BBB function, Adropin not only improves the functional status of endothelial cells, but also interacts with signaling pathways that reinforce BBB integrity. It promotes endothelial cell proliferation and migration, hence enhancing capillary angiogenesis [158]. Furthermore, Adropin promotes angiogenesis by activating the VEGF signaling pathway and inhibits the expression of pro-inflammatory cytokines TNF-α and IL-6 [159]. It reduces matrix metalloproteinase MMP-9 levels and sustains the expression of tight junction proteins such as occludin, ZO-1, and claudin-5, preserving BBB integrity [160]. In ischemic stroke models, adropin activates the eNOS-PI3K-AKT-ERK1/2 signaling axis, increasing phosphorylated eNOS levels and reducing stroke-induced BBB permeability [156]. In subarachnoid hemorrhage models, it decreases BBB permeability and albumin leakage. Its neuroprotective effects are mediated through the Notch1/Hes1 signaling pathway [161].

Beyond its vascular benefits, Adropin exerts metabolic effects that may alleviate BBB damage. By inhibiting pyruvate dehydrogenase kinase-4, Adropin reduces pyruvate dehydrogenase complex (PDHC) acetylation, thereby restoring PDHC activity and promoting oxidative decarboxylation of pyruvate by Gpr19-P44/42 MAPK pathway [162]. It is noteworthy that Adropin has been shown to prevent increases in endothelial cell permeability and to reduce macrophage transmigration across the endothelial monolayer. To date, it remains unclear whether Adropin can cross the BBB. Based on its molecular size and lipophilic properties, it appears unlikely that Adropin can readily diffuse from the systemic circulation into the CNS [160, 163]. Consequently, it may only exert its effects under conditions of BBB disruption, a possibility that warrants further investigation.

Adropin mediated AKT activation reduces FOXO3a levels, thereby inhibiting oxidative stress and mitochondrial damage [164]. The dynamic regulation of the AKT signaling pathway holds significant pathophysiological relevance in neurodegenerative diseases, as its activity state influences neuronal survival and resistance to proteotoxic stress through multiple mechanisms. In PD models, pathological changes are characterized not only by the selective loss of specific dopaminergic neurons but also by decreased activity at critical AKT phosphorylation sites [165]. Adropin exerts neuroprotective effects by activating AKT, thereby supporting dopaminergic neuron survival [165]. This provides a theoretical basis for therapeutic strategies aimed at enhancing AKT activity. In the pathological mechanisms of Huntington’s disease, the neurotoxicity induced by mutant huntingtin’s protein can be antagonized by AKT phosphorylation, which involves the modification of key regulatory factors in apoptotic signaling pathways, such as GSK3β and FOXO1 [165]. Furthermore, activated AKT reduces nuclear inclusions of huntingtin protein, mitigates abnormal protein aggregation, and delays neuronal functional decline. Therefore, the regulation of the AKT signaling pathway by Adropin may influence neuronal survival in neurodegenerative diseases [165].

Notably, while numerous studies have highlighted the significant role of Adropin in protecting against BBB damage, research on the direct molecular associations between Adropin and the core pathological mechanisms of AD remains relatively limited. Therefore, future studies should further investigate the potential molecular mechanisms of Adropin in regulating key pathological processes of AD, particularly its effects on Aβ clearance, neuroinflammation inhibition, and synaptic function protection. This would provide stronger theoretical support for developing Adropin-targeted therapeutic strategies for AD.

### ApoJ

ApoJ, or Clusterin, is a multifunctional heterodimeric glycoprotein consisting of disulfide-linked α and β subunits [166]. ApoJ is mainly synthesized in the liver and secreted into the blood. As a component of HDL cholesterol, ApoJ can directly participate in cholesterol metabolism and regulate oxidative stress in LDL [167]. Acting as an extracellular chaperone, ApoJ binds to the hydrophobic regions of unfolded proteins, preventing protein aggregation and mitigating cellular damage by reducing oxidative stress [166].

In an in vitro BBB model, it was discovered that when Aβ_1−40_ forms a complex with recombinant ApoJ, the efflux efficiency of Aβ from the brain to the blood side is significantly enhanced [168, 169]. This process is mediated by LRP-1 and can be inhibited by receptor-associated protein [168]. Other studies have also confirmed that the brain uptake of the sAβ_1−40_-ApoJ complex is over 100-fold greater than that of the sAβ_1−40_-ApoE complex. Radiolabeled iodine experiments demonstrated that following carotid artery injection, sAβ_1−40_-ApoJ reached 10.2% of its plasma perfusion concentration within just 10 min, exhibiting exceptionally high trans-endothelial transport efficiency. Its brain uptake was 37 times greater than that of the vascular space marker sucrose [170]. Notably, the interaction between recombinant ApoJ and Aβ not only facilitates Aβ translocation across the BBB but also inhibits Aβ_1−40_ and Aβ_1−42_ fibril aggregation, as demonstrated by thioflavin-T binding assays [168].

Ko et al. through an analysis of human CSF samples, observed a 23% reduction in CSF ApoJ concentrations in patients with mild cognitive impairment (MCI) compared to control groups, with a similar declining trend in AD patients [13]. Additionally, cholesterol efflux capacity (CEC) in the CSF of MCI patients was significantly reduced compared to healthy controls, whereas ApoJ has been shown to promote cholesterol efflux from brain cells and inhibit Aβ deposition. Interestingly, although carriers of the ApoE ε4 genotype exhibit reduced CSF ApoE levels, no significant association was found between ApoE ε4 and CEC [13]. Building on the molecular interactions between ApoJ and Aβ, additional studies have explored its therapeutic potential using materials science approaches. For instance, Retana et al. assembled purified ApoJ with phospholipids into recombinant HDL nanodiscs [169]. These nanodiscs effectively inhibited Aβ_1−40_ and Aβ_1−42_ fibrillization and significantly enhanced CEC in macrophages [169].

ApoJ synthesized by astrocytes contributes significantly to its CNS pool, although serum ApoJ levels have been proposed as a potential biomarker for the conversion from MCI to dementia [171]. Animal studies further support its therapeutic potential; peripheral administration of recombinant human ApoJ reduces insoluble Aβ_42_ levels in APP23 mouse brains [169]. Furthermore, peripheral ApoJ supplementation mitigated hippocampal neuronal loss, lowered IL-17 inflammatory cytokine release, and improved microglial phagocytosis of Aβ deposits [172, 173]. ApoJ, when combined with simvastatin, further enhanced the clearance of Aβ by endothelial cells and reduced central Aβ accumulation [174].

However, conflicting results have also been reported. Histological analyses of the temporal cortex in AD patients revealed co-localization of ApoJ in 29% of brain parenchymal Aβ deposition areas. Remarkably, 71% of ApoJ-positive amyloid plaques were surrounded by dystrophic neurites marked by phosphorylated tau protein, while 64% of tau-positive neurofibrillary plaques showed ApoJ immunoreactivity. In vitro experiments further demonstrated that exposure to exogenous ApoJ significantly upregulated total tau expression levels in mouse brain tissue and primary neuronal cells while enhancing tau phosphorylation. These findings suggest that ApoJ contributes to disease progression not only by stabilizing amyloid fibril structures and accelerating plaque maturation but also by modulating post-translational modifications of tau protein, thereby exacerbating neuronal pathological damage [175]. Furthermore, genome-wide association studies (GWAS) have linked elevated ApoJ levels with a higher risk of late-onset AD, possibly due to SNP polymorphisms in rs2279590 and rs11136000 [176]. These genetic variants may impair astrocytic responses to Aβ plaques and exacerbate axonal and neuronal damage [177, 178]. Moreover, studies involving ApoJ gene knockout have shown reduced Aβ fibril formation and prevention of Aβ-induced neuronal death, suggesting that ApoJ might also contribute to AD pathology under certain conditions. The reasons for this discrepancy need to be further studied.

In other neurodegenerative diseases, ApoJ similarly plays a regulatory role. A study based on the BioFIND cohort found that in patients with PD [179], the binding of CSF α-syn to ApoJ and ApoE exhibited a specific upregulation. In stark contrast, the concentrations of ApoA-I and ApoJ in the peripheral circulatory system were significantly lower compared to healthy controls [179]. This brain-peripheral inverse regulation phenomenon may reflect either selective transport dysfunction across the BBB or systemic energy metabolism imbalance.

### SHBG

The association between sex differences and AD has become an area of increasing research interest. In females, reduced 17β-estradiol levels, a type of estrogen, are closely associated with cognitive decline. In males, early testosterone depletion has been identified as a risk factor for early-onset AD, with lower testosterone levels frequently linked to cognitive impairment [180].

SHBG is a liver-synthesized glycoprotein with a molecular weight of approximately 90 kDa and is critical for regulating the bioavailability of sex hormones [181]. In plasma, only 1–3% of sex hormones are biologically active in their free form, while approximately 38% of estrogens and 44% of androgens are bound to SHBG [182]. This binding not only protects sex hormones from rapid degradation but also maintains their biological activity and ensures stable blood hormone levels [183].

The ability of SHBG to cross the BBB remains unclear. Previous research has generally held that SHBG does not cross the BBB [184]. However, other studies that measured the CSF-to-plasma ratio have found that the CSF/plasma ratio for SHBG remains constant. In patients with an intact blood-CSF barrier, measured SHBG levels were 0.139 nmol/L in females and 0.083 nmol/L in males. In contrast, among 18 cases with impaired blood-CSF barrier function, the correlation between CSF SHBG levels and total protein was similar to that observed for albumin and IgG. These findings suggest that under pathological conditions, SHBG may have the ability to cross a compromised BBB [185].

Evidence from animal models highlights the impact of testosterone depletion on mitochondrial function in AD. In castrated APP/PS1 mice, reduced testosterone levels were associated with decreased mitochondrial DNA copy number in the hippocampus and downregulation of key mitochondrial regulators, including when PGC-1α and Nrf-1 [186, 187]. These changes disrupted mitochondrial dynamics, leading to structural abnormalities, such as disorganized cristae, and excessive ROS production [187]. The resulting oxidative stress accelerated neuronal damage and degeneration, ultimately promoting the progression of AD [188]. Human studies corroborate these findings. A meta-analysis by Lv et al. indicated that lower plasma testosterone levels in older men are linked to a higher risk of AD, suggesting that testosterone deficiency is a potential risk factor for cognitive impairment [189]. However, the association of testosterone and its carrier protein SHBG with cognition requires further elucidation. While reduced SHBG levels are associated with hepatic steatosis and hyperinsulinemia, their direct effects in AD pathogenesis remains controversial [190]. Given SHBG’s dual role in regulating sex hormones and energy metabolism, its potential involvement in AD warrants additional investigation.

The potential association between SHBG and neurodegenerative diseases has been investigated in several studies employing Mendelian randomization methods to explore the impact of genetically predicted sex hormone levels on disease risk. Current evidence suggests that elevated genetically predicted sex hormone concentrations may be positively associated with the risk of MS and ALS. This pattern of association implies that circulating SHBG levels may play a specific role in the pathogenesis of neurodegenerative disorder [191]. Consequently, the exact role of SHBG in neurodegenerative diseases and its clinical translational value require further validation through rigorously designed studies. In particular, it is important to address reverse causality and potential confounding factors to advance understanding in this area.

### Others hepatokines

Hepatokines constitute a diverse and expanding family. While eight hepatokines have been discussed above, ongoing research continues to identify new members, further expanding our understanding of their functional diversity.

Follistatin (FST) is a glycoprotein predominantly secreted by the liver and expressed in various tissues, functioning to inhibit TGF-β superfamily members. This inhibition is associated with the regulation of reproductive functions and the promotion of skeletal muscle growth [192, 193]. Specific knockout of FST reduces hippocampal neurogenesis in mice, impairing spatial cognition and long-term enhancement [194]. However, FST can improve IR and inhibit inflammation through the interaction of TGF-β family members [195], and TGF-β mediates the production-distribution of amyloid [196], so it is reasonable to speculate that FST may play a mediating role in influencing AD through TGF-β.

Sparc-related modular calcium-binding protein 1 (SMOC1) has been identified as a glucose-responsive hepatokine that modulates dynamic glucose homeostasis by enhancing glycaemic control and insulin sensitivity independently of insulin secretion [197]. Increasingly, however, SMOC1 has also been implicated in AD: a comprehensive analysis based on large-scale mass spectrometry, encompassing 14,513 brain proteins and 34,173 phosphorylation sites, revealed the temporal dynamics of 173 proteins across 17 critical pathways involved in AD progression [198]. Among these proteins, SMOC1 was identified as a central hub with significant RNA-dependent expression characteristics, validated in two independent cohorts. This finding suggests that its regulation may involve post-transcriptional modifications or interactions with non-coding RNAs [199]. Integration of autosomal dominant AD patient CSF proteomics with brain tissue pathology identified SMOC1 as being abnormally elevated in the CSF nearly 30 years before the onset of cognitive symptoms, marking it as the earliest known biomarker of AD [200, 201]. Notably, this elevation occurs well before the pathological accumulation of conventional biomarkers such as Aβ and tau, highlighting its potential for early disease detection. SMOC1 is widely distributed in AD-vulnerable brain regions, including the temporal cortex, hippocampus, and frontal cortex [200]. Its cerebral levels show a positive correlation with amyloid plaque burden, with SMOC1 co-localizing on plaque surfaces in late-stage AD. However, the proportion of co-localization progressively increases from the MCI stage to advanced AD [202]. Molecular interaction studies further revealed that SMOC1 forms stable complexes with Aβ as early as the MCI stage, with binding strength intensifying as the disease progresses [200]. Functionally, SMOC1 delays Aβ fibril maturation in a dose-dependent manner, resulting in aberrant fibril morphology and an extended aggregation lag phase [200]. In contrast, its interaction with phosphorylated tau becomes significantly activated only in late-stage AD, suggesting that SMOC1 is involved in distinct pathological processes at different stages of the disease [200].

Soluble epoxide hydrolase (sEH), a 62.5 kDa bifunctional enzyme encoded by the EPXH2 gene on chromosome 8, is widely distributed across the brain. It is predominantly found in neuronal soma and glial cells [203]. The enzyme comprises two active regions: the C-terminal domain responsible for epoxide hydrolase activity and the N-terminal domain with phosphatase functionality, both of which are essential for the regulation of brain cholesterol metabolism and inflammatory responses [204]. Elevated levels of sEH have been identified in the brains of postmortem AD patients and in APP/PS1 transgenic mouse models, suggesting its involvement in AD pathogenesis [203]. Suppressing hepatic sEH activity through Ephx2 genetic ablation results in increased plasma concentrations of 14,15-epoxyeicosatrienoic acid [205]. This metabolite not only traverses the BBB to inhibit Aβ aggregation via direct interaction but also facilitates Aβ clearance mediated by microglia through the ApoE-TREM2 signaling pathway [205].

Small-molecule inhibitors like TPPU have demonstrated therapeutic potential in preclinical AD studies [206]. In 5×FAD mouse models, these approaches reduce glial activation and decelerate Aβ-related pathological progression [206, 207]. Transcriptomic studies of cerebrovascular smooth muscle cells treated with sHE inhibitor in DM-ADRD rat models revealed significant enrichment of the cell contractile pathways, as identified by KEGG analysis [208]. The impact of TPPU on vascular dynamics is concentration-dependent: low doses enhance vascular smooth muscle cell-mediated myogenic responses, while higher doses improve endothelium-dependent vasodilation [208]. Additionally, sHE inhibitor mitigates the generation of ROS and mitochondrial superoxide in the cerebral vasculature of AD models [208]. These effects indicate that inhibition of sEH activity effectively alleviates cognitive decline in AD, suggesting that some liver products may regulate amyloid pathology and cerebrovascular damage in AD through the circulatory system or neural pathways [209].

In summary, hepatokines are emerging as crucial mediators of liver-brain communication, influencing both systemic metabolism and neurological function. Their involvement in AD-related processes highlights the importance of further research to elucidate their roles and therapeutic potential within the context of metabolic regulation and neurodegeneration.

## Interlinked pathways: metabolic disorders and AD

**Fig. 3:**
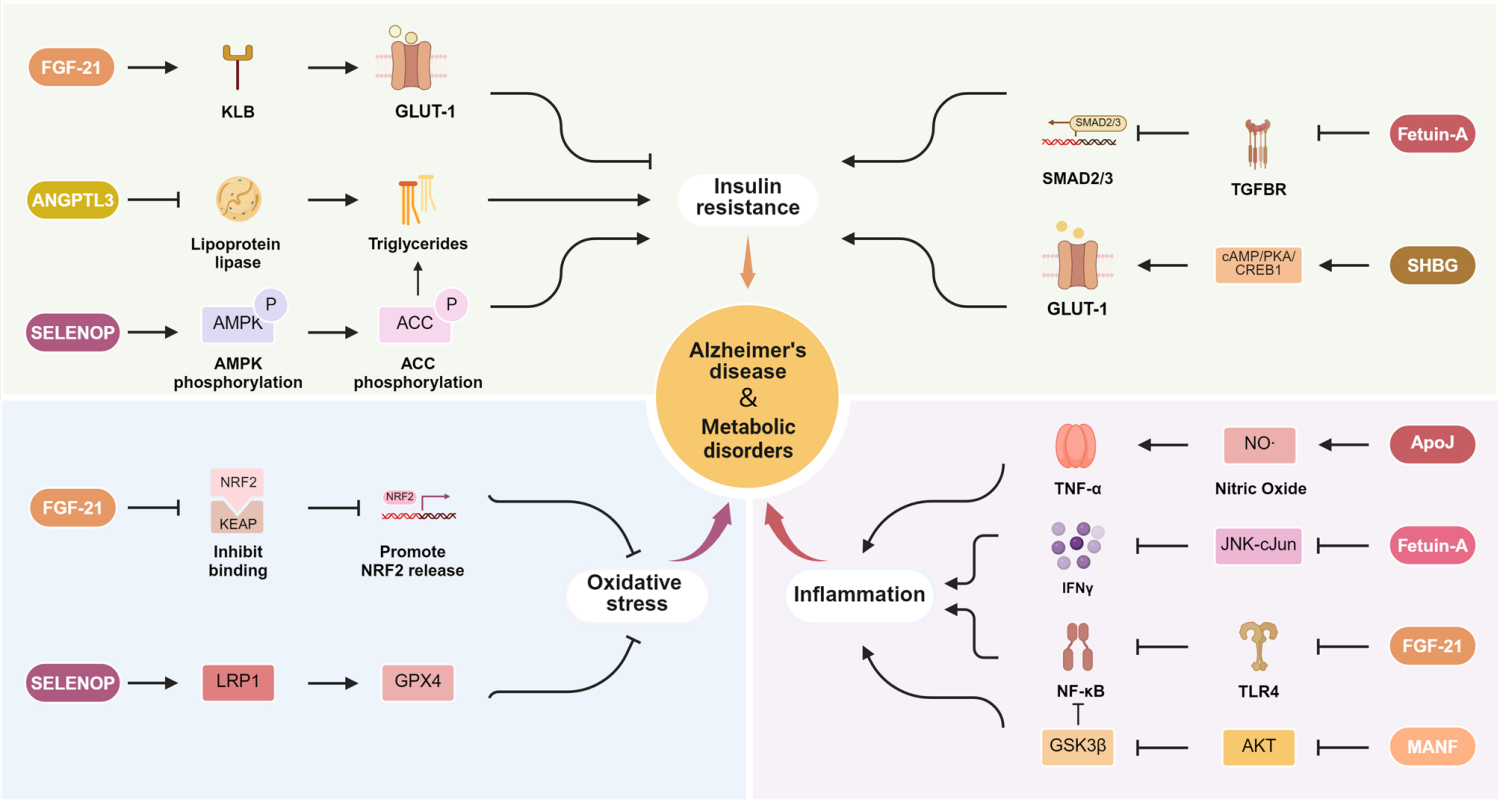
The bridging role of hepatokines in AD and metabolic disorders. This figure highlights the central role of hepatokines in linking AD and metabolic disorders through mechanisms involving IR, neuroinflammation, and oxidative stress. In IR, FGF-21 binds to KLB receptors to promote GLUT-1-mediated glucose uptake and alleviate IR, while ANGPTL3 increases blood TG levels by inhibiting LPL activity. SELENOP suppresses AMPK phosphorylation, reducing ACC phosphorylation, thereby enhancing fatty acid synthesis and exacerbating IR. Reduced levels of Fetuin-A attenuate TGFBR signaling in pancreatic islets, inhibit SMAD2/3 phosphorylation, and regulate insulin secretion. SHBG enhances IR by regulating glucose transporter expression through the cAMP/PKA/CREB1 signaling pathway. In neuroinflammation, ApoJ stimulates microglia, enhancing reactive nitrogen intermediates and pro-inflammatory cytokines like TNF-α. Concurrently, Fetuin-A facilitates macrophage migration and polarization via the JNK-c-Jun-IFN-γ–JAK2–STAT1 pathway, intensifying adipose tissue inflammation. Conversely, FGF-21 inhibits NF-κB signaling via the TLR4 receptor, and MANF provides neuroprotection by suppressing NF-κB during ER stress and activating the AKT/GSK3β-Nrf2 axis. In oxidative stress, FGF-21 activates FGFR1 to destabilize the Keap1-Nrf2 complex, enabling Nrf2 to initiate the antioxidant response, while SELENOP binds to LRP-1 to maintain Gpx4 activity and prevent lipid peroxidation

As a central organ of metabolism, the liver is essential in linking metabolic disorders, such as IR and dysregulated glucose and lipid metabolism, to the pathogenesis of AD. By modulating systemic metabolic homeostasis, the liver acts as a key intermediary in the progression of metabolic syndrome and its neurological complications. The intricate interplay between metabolic disorders and AD involves shared mechanisms, including the disruption of insulin signaling, chronic neuroinflammation, and increased oxidative stress. Hepatokines, metabolic regulatory factors secreted by the liver, serve as important modulators in this relationship. These hepatokines exhibit diverse and sometimes paradoxical roles, with certain hepatokines exacerbating peripheral metabolic dysfunction while potentially mitigating AD pathology within the CNS. This section examines the multifaceted contributions of hepatokines to the pathogenesis of metabolic disorders and AD, focusing on their regulatory mechanisms and therapeutic implications (Figure.3).

### Insulin resistance

The brain’s high sensitivity to insulin is underscored by the abundance of insulin receptors in the hippocampus [210], where insulin signaling regulates synaptic plasticity, neurogenesis, and cognitive function through pathways such as PI3K/AKT, GSK3β, and mTOR [211]. Disruption of these pathways due to IR impairs synaptic plasticity, promotes neurodegeneration, and increases the risk of AD. Specifically, IR-induced GSK3β activation promotes tau hyperphosphorylation and neurofibrillary tangle formation [212]. Knockout models of insulin receptors demonstrate reduced AKT and GSK3β phosphorylation, leading to enhanced neuronal apoptosis [213]. Concurrently, impaired MAPK/ERK and CREB/BDNF signaling disrupt synaptic plasticity and neurotransmission [214]. Additionally, insulin signaling aids in Aβ clearance and shields neurons from the toxicity of misfolded proteins [215].

Hepatokines, including FGF-21, ANGPTL3, SELENOP, Fetuin-A, and SHBG, modulate IR through diverse mechanisms, exhibiting dichotomous effects between the periphery and CNS. In conditions of IR and overnutrition, hepatocytes increase FGF-21 secretion to enhance insulin sensitivity and alleviate metabolic stress [216]. Interestingly, the methylation of the FGF-21 gene declines prior to the onset of overt metabolic abnormalities, suggesting that this epigenetic change may represent an adaptive response by the liver to maintain glucose homeostasis during the early stages of metabolic dysregulation [216]. However, mutations in FGF-21 receptors, including FGFR1 and KLB, impair FGF-21 signaling, reducing GLUT-1 and GLUT-4 mediated glucose uptake, which exacerbates IR. The inflammatory milieu associated with IR further complicates this adaptive mechanism. Chronic inflammation triggers the release of pro-inflammatory cytokines such as TNF-α, which in turn suppresses FGF-21 expression [217]. This suppression establishes a self-reinforcing cycle where inflammation exacerbates IR by diminishing the regulatory capacity of FGF-21 [218]. Besides, there are also studies based on the integrated analysis of ^1^H nuclear magnetic resonance metabolomics and dynamic ^13^C isotope tracer technology showing that FGF-21 intervention effectively remodeled glucose metabolism in the brain, facilitating the conversion of [^1–13^ C]-glucose to the key neurotransmitter precursor and energy metabolism intermediate [^3–13^ C]-lactate while restoring metabolic homeostasis to the γ-aminobutyric acidergic system [219]. Despite these challenges, elevated FGF-21 levels have been shown to consistently decrease hepatic steatosis, and improve IR in metabolic disorders caused by high-fat diets [220]. This highlights the dual role of FGF-21 as a compensatory mechanism in early metabolic stress and a therapeutic target for advanced metabolic disorders.

Elevated ANGPTL3 levels are frequently observed in metabolically disturbed obese populations and positively correlate with various IR markers, highlighting its role as a hepatokine implicated in IR pathogenesis [221]. GWAS have identified mutations in the ANGPTL3 gene, specifically rs1748197 and rs12130333, which are associated with reduced C-peptide levels, further supporting the involvement of ANGPTL3 in the development of IR [222]. Mechanistically, ANGPTL3 exerts its effects on lipid metabolism and IR through the inhibition of LPL activity. This inhibition leads to elevated circulating levels of VLDL and TG, impairing lipid clearance and metabolic balance [223]. The increase in VLDL and TG subsequently contributes to higher LDL levels, thereby exacerbating systemic IR [135]. Furthermore, ANGPTL3 interacts with ApoA-I in HDL, which obstructs the reverse cholesterol transport capability of HDL, compounding its impact on lipid metabolism [135].

Although ANGPTL3 exacerbates metabolic derangement and AD, its central and peripheral effects, as with FGF-21, are consistent. SELENOP, however, has an opposite effect between the CNS and peripheral disease. In patients with T2DM and other metabolic abnormalities, elevated SELENOP levels exacerbate IR and reduce the efficiency of insulin transport into the CNS, thereby compounding metabolic dysfunction and possibly contributing to neurodegenerative processes [224]. Mechanistically, SELENOP promotes IR by inhibiting the phosphorylation of Acetyl-CoA carboxylase (ACC), a critical enzyme in lipid metabolism. This inhibition occurs through the suppression of AMPK phosphorylation, leading to increased fatty acid synthesis and reduced metabolic flexibility [225]. Importantly, dietary fatty acid composition significantly influences hepatic SELENOP expression. Polyunsaturated fatty acids (PUFAs) decrease hepatic SELENOP expression, potentially mitigating its detrimental metabolic effects. In contrast, medium- and long-chain saturated fatty acids, such as palmitic acid, increase SELENOP expression. This upregulation is mediated by the binding of hepatic nuclear factor 4 alpha (Hnf4α) to the SELENOP promoter, linking dietary composition to hepatic SELENOP regulation and systemic metabolic health [226]. While elevated SELENOP levels in the periphery are detrimental, increased CNS SELENOP may confer protective effects. This may be due to the fact that in metabolic disorders, SELENOP inhibits the burst of ROS required for normal insulin signaling due to the powerful reducing effect of SELENOP, so this condition is also known as reductive stress [227].

Like SELENOP, elevated CNS Fetuin-A levels may play a neuroprotective role, but it can aggravate IR in peripheral metabolism [228]. Elevated peripheral Fetuin-A levels are closely associated with hepatic steatosis and IR [229]. In mouse models, peripheral supplementation of Fetuin-A, combined with high-fat diets, worsens pancreatic islet organization and impairs insulin secretion [230]. Conversely, silencing the Fetuin-A gene improves islet cell viability and glucose-stimulated insulin secretion [231]. Mechanistically, decreased Fetuin-A levels alleviate growth factor-beta receptor (TGFBR) signaling in pancreatic islets, and inhibit phosphorylation of SMAD2/3. This impairment hinders β-cell functional maturation and insulin release [232].

SHBG is inversely correlated with IR, playing a critical regulatory role in glucose metabolism and insulin signaling. Reduced SHBG levels are consistently associated with reduced diminished activity in the PI3K/AKT signaling pathway, a central mediator of insulin action [233]. In insulin-resistant cell models, SHBG expression is downregulated, which leads to increased GLUT1 expression along with decreased GLUT3 and GLUT4 levels [234]. Conversely, overexpression of SHBG reverses these effects, indicating that SHBG may mitigate IR by regulating glucose transporter expression via the cAMP/PKA/CREB1 signaling pathway [235]. In addition, low SHBG levels may reflect increased androgen binding, which reduces circulating and central androgen levels and potentially increases central IR, contributing to neurological damage [181].

Beyond its role in IR, certain hepatokines also regulate glucose metabolism through alternative pathways. Liang et al. found that intracerebroventricular injection of recombinant FGF-21 protein promoted hypothalamic corticotropin-releasing hormone expression and activated the hypothalamic-pituitary-adrenal axis in mice, which rebounded plasma corticosterone levels to 85% of those of the wild-type and reversed hepatic glucose allosteric defects [12]. Additionally, inhibition of ANGPTL3 was shown to suppress the expression of key gluconeogenic enzymes, particularly glucose-6-phosphatase, thereby attenuating hepatic gluconeogenesis [236].

In conclusion, certain hepatokines hold significant potential due to their dual role in improving both IR and AD. Since IR is known to influence AD pathology, the ability of these hepatokines to impact both conditions suggests a multifaceted therapeutic potential for addressing metabolic and neurological dysfunctions. While the roles of various hepatokines in IR are increasingly recognized, inconsistencies and paradoxes remain regarding their effects across peripheral and CNS.

### Oxidative stress and mitochondrial dysfunction

Mitochondria are not only the primary site of ROS production but also major targets of oxidative stress. In the CNS, mitochondrial function is essential for sustaining neuronal energy balance, thereby supporting synaptic activity and neuron viability. The synthesis of ATP is impaired by disruption of mitochondrial membrane potentials, reduced activity of respiratory chain enzymes, and dysregulation of intracellular calcium homeostasis. These dysfunctions impair cellular energy production and promote oxidative damage [237]. The brains of AD patients exhibit a significantly elevated rate of mitochondrial DNA mutations compared to age-matched healthy individuals [238].

Oxidative stress is both a cause and a result of metabolic disorders. Metabolic dysregulation-induced oxidative stress may cause lipid peroxidation, DNA damage, and protein dysfunction [239]. An improvement in metabolism is often accompanied by a reduction in oxidative stress. Nrf2 is a crucial regulator of antioxidant responses. Nrf2 binds to antioxidant response elements (AREs) to enhance the expression of proteins essential for maintaining cellular redox balance [240, 241]. During mitochondrial dysfunction, Nrf2 is typically inhibited by Keap1, which stabilizes the complex and prevents Nrf2 activation. FGF-21 activates FGFR1, leading to the destabilization of the Keap1-Nrf2 complex. This process releases Nrf2, enabling its nuclear translocation to initiate the antioxidant response [242]. In AD models, Nrf2 deficiency exacerbates oxidative stress, leading to increased lipid peroxidation and DNA damage, particularly in astrocytes, which accelerates neurodegeneration and inflammation. These findings suggest that FGF-21 reduces oxidative stress via Nrf2, indicating potential crosstalk between metabolic disorders and AD. Additionally, FGF-21 reduces Keap1-Nrf2 binding, thereby inhibiting oxidative stress and preserving BBB integrity [243]. Additionally, in metabolic disorders, FGF-21 alleviates mitochondrial stress by regulating the ERK1/2 pathway and modulating activating transcription factor 5 (ATF5) and C-Myc expression [244]. Furthermore, FGF-21 treatment notably enhanced antioxidant enzyme activities in both serum and brain tissues of mice [245]. FGF-21 enhances mitochondrial deacetylase SIRT3 expression via AMPK-mediated FOXO3 phosphorylation, safeguarding against lipotoxicity-induced mitochondrial dysfunction and oxidative stress [246].

SELENOP maintains the activity of glutathione peroxidase 4 (Gpx4) by binding to LRP-1, preventing lipid peroxidation and ferroptosis, both of which contribute to neurodegeneration in AD [15]. SELENOP not only delivers Se to neurons but also interacts with misfolded Aβ and tau proteins, alleviating oxidative stress and preventing tau hyperphosphorylation by modulating metal ions levels in synapses [246, 247]. However, SELENOP levels in the peripheral circulation are inversely correlated with metabolic syndrome, visceral and subcutaneous abdominal fat, and liver fat content [155]. Moreover, a U-shaped relationship exists between plasma Se concentration and metabolic syndrome: peak antioxidant protection occurs at the curve’s nadir. However, the antioxidant benefit of SELENOP can be obscured under conditions of reductive stress [246].

In conclusion, mitochondrial dysfunction, oxidative stress, and metabolic disturbances significantly drive AD progression. Hepatokines may offer therapeutic benefits by mitigating oxidative stress and restoring mitochondrial function.

### Inflammation

Inflammation is a key pathological mechanism in AD, with neuroinflammation as a specific immune response in the CNS to internal and external stimuli. Microglia reduce tau phosphorylation in early AD by clearing Aβ plaques and secreting anti-inflammatory cytokines [248]. The accumulation of Aβ leads to the overactivation of microglia and astrocytes, triggering release of pro-inflammatory cytokines, such as IL-1 and TNF-α, that sustain chronic neuroinflammation [249]. Microglia also recruit astrocytes through the complement cascade, amplifying inflammation. Consequently, modulating this neuroinflammatory cascade remains a key therapeutic target in AD [250].

Metabolic disorders are also strongly linked to inflammation, and their impact on the CNS are partially mediated through inflammatory pathways. For instance, NF-κB, a crucial mediator of inflammation, induces transcription of pro-inflammatory factors, leading to synaptic dysfunction and neuronal loss. NF-κB activation further triggers microglial activation and oxidative stress, contributing to synaptic impairment [212]. High-fat dietary interventions could activate NF-κB through the CD14-TLR4-MD-2 complex, impairing glial function [250]. Besides, in models of high-fat diets, free fatty acid receptor 4 (FFAR4) also worsens neuroinflammation through NF-κB and IFN-β signaling pathways [251]. Additionally, the MAPK pathway drives glial morphological changes and release of pro-inflammatory cytokines, promoting neuroinflammation and synaptic dysfunction [252]. Inhibition of p38-MAPK has shown potential to alleviate cognitive impairment in metabolic disorders by reducing inflammation [252, 253].

FGF-21 inhibits NF-κB signaling via the TLR4 receptor, protecting neurons from Aβ-induced apoptosis and reducing neuroinflammation by downregulating NF-κB, IL-6, and IL-8 [254]. Caloric restriction could upregulate FGF-21 and inhibit tau hyperphosphorylation by modulating AMPK and mTOR pathways [255]. Besides, in ketogenic dietary interventions, FGF-21 reduces IL-1β secretion by macrophages, further alleviating systemic inflammation and metabolic dysfunction [256].

MANF offers neuroprotection by inhibiting NF-κB during ER stress and activating the AKT/GSK3β-Nrf2 axis, reducing TNF-α and IL-6 and slowing neurodegeneration [257]. Furthermore, ApoJ can promote the export of cholesterol from foam cells and exhibit anti-inflammatory effects by interacting with inflammation-related proteins such as C-reactive protein [258]. However, Fetuin-A, elevated in conditions of obesity and high-fat diets, promotes macrophage migration and polarization via the JNK-c-Jun-IFN-γ–JAK2–STAT1 pathway, leading to adipose tissue inflammation [259]. Hyperglycemia further increases Fetuin-A expression, worsening metabolic disturbances via the ERK1/2 pathway, although its role in Aβ clearance warrants further study [260].

In conclusion, neuroinflammation is pivotal in the onset and progression of AD, with its interaction with metabolic disorders worsening the pathological process. Certain hepatokines exhibit neuroprotective effects by suppressing inflammation and regulating metabolic pathways, potentially offering novel therapeutic strategies. However, some hepatokines may have dual roles, exacerbating the pathological process in certain conditions while providing protective effects in others.

## Clinical and therapeutic applications

Hepatokines hold significant promise for diagnosing and treating AD. Altered levels of hepatokines such as FGF-21, SHBG, and MANF in AD patients suggest their utility as diagnostic biomarkers and therapeutic targets. The modulation of hepatokine levels by lifestyle factors such as exercise and diet further underscore their relevance in AD management. Table 1 summarizes the current research on hepatokines in clinical diagnosis and therapy.

### Hepatokines as diagnostic biomarkers

The diagnosis of AD remains a multifaceted challenge, requiring comprehensive assessments that combine patient history, neuroimaging, biomarker evaluation, and risk factor analysis [261]. Current diagnostic tools, such as MRI and PET, are valuable for detecting pre-symptomatic changes and visualizing neuropathological alterations [262]. Despite these advances, the complexity of AD, characterized by its heterogeneity and dynamic biomarker profiles, necessitates the development of more accessible and cost-effective diagnostic approaches. Blood-based biomarkers have gained increasing attention for their convenience and minimally invasive nature in predicting, diagnosing, and monitoring AD. Among these, hepatokines represent an emerging class of biomarkers with significant diagnostic potential.

Among the hepatokines, SHBG and Fetuin-A have been the most extensively studied. Elevated SHBG has been positively correlated with AD risk in older cohorts, suggesting its potential as a predictive biomarker [263]. However, large-scale, multi-center studies are needed to confirm its diagnostic value. Similarly, preliminary research indicates that Fetuin-A levels exhibit an inverse relationship with inflammatory markers, such as TNF-α, and a positive association with cognitive performance. Higher levels of Fetuin-A correlate with improved scores on the MMSE and Trails B scales, while lower levels are associated with dementia [264]. Despite these promising findings, longitudinal studies are needed to establish its reliability as a diagnostic marker.

In addition, emerging biomarkers like FGF-21 and MANF have shown potential for early detection of AD. FGF-21 has been found at significantly lower levels in AD patients compared to long-lived older adults [265]. This suggests a potential diagnostic role, though its sensitivity and specificity in detecting early AD require further investigation. Neuropathological studies have found MANF overexpression in the inferior temporal gyrus of both preclinical and clinical AD patients. This finding highlights its possible involvement in early neurodegeneration, although its validation as a blood-based biomarker remains incomplete [266]. Additionally, ApoJ has shown promise in predicting the progression from MCI to AD [171].

### Lifestyle interventions

Hepatokines may offer therapeutic benefits in AD by modulating metabolism, inflammation, and neuroprotection. Exercise and dietary interventions, which influence the expression of hepatokines, may synergize with hepatokines-targeted therapies to mitigate AD progression. This section reviews how these interventions affect hepatokines and their therapeutic potential in managing AD and metabolic disorders.

### Exercise

Exercise is a well-established non-pharmacological intervention for improving metabolic and neurological health, in part through its influence on hepatokine expression. Different types, intensities, and durations of exercise elicit distinct effects on key hepatokines, including FGF-21, SELENOP, Fetuin-A, and ApoJ.

Aerobic exercise enhances FGF-21 sensitivity through the upregulation of the adiponectin receptor [267]. In obese rats, continuous exercise training (CET) increases liver FGF-21 expression, while high-intensity interval training (HIIT) promotes mitochondrial lipid oxidation and hepatic fatty acid uptake enzyme activity. Both exercise regimens reduce hepatic lipid accumulation and body weight [268]. FGF-21 also enhances glucose uptake during muscle activity by activating the P2Y/PI3K/AKT/mTOR pathway, further underscoring its role in exercise-induced metabolic benefits [269]. In sedentary mice, hepatic FGF-21 mRNA and protein levels were notably elevated compared to active controls, with acute exercise causing a temporary increase [270]. Furthermore, after exercise, liver FGF-21 levels rose within 1 h, but dropped 24 h later, suggesting that exercise-induced changes in FGF-21 are time-dependent [271].

The effects of exercise on SELENOP levels are less consistent. Regular exercise was associated with lower SELENOP levels and increased mitochondrial DNA copy number in leukocytes [272]. In contrast, another study reported that HIIT significantly elevated serum SELENOP levels compared to sedentary and moderate exercise groups, increasing SELENOP levels by 84% with HIIT and by 33% with moderate exercise, suggesting that exercise intensity may play a determining role in regulating SELENOP levels [273].

Exercise-induced changes in Fetuin-A levels are dynamic. Moderate-intensity exercise temporarily increases serum Fetuin-A levels in obese individuals, but these levels decline significantly 24 h post-exercise, correlating with improved insulin sensitivity [274]. Long-term exercise program has been demonstrated to lower Fetuin-A levels in plasma and subcutaneous adipose tissue among both diabetic and non-diabetic obese individuals, although the precise regulatory mechanism remains unclear [275]. Furthermore, combined aerobic and resistance training decreases circulating ApoJ levels. In postmenopausal women with diabetes, ApoJ levels dropped by 26.3% and 19.4% after 8 and 12 weeks of exercise, respectively, showing a strong correlation with improved IR [276].

Overall, as metabolic impairments and mitochondrial dysfunction are key drivers of AD pathology, exercise-mediated hepatokine regulation offers a viable strategy for preventing or slowing AD progression. However, the specific outcomes depend on exercise type, intensity, and duration, necessitating further research to standardize protocols and elucidate mechanisms.

### Diet

Diet plays a critical role in hepatokine regulation and systemic metabolic homeostasis, with specific dietary patterns influencing the expression of FGF-21, MANF, SHBG, Fetuin-A, and SELENOP. This section examines how different dietary patterns influence AD and metabolic diseases by hepatokines.

FGF-21 is highly sensitive to dietary composition. Low-protein, high-energy diets significantly elevate FGF-21 levels, which in turn regulate hypothalamic appetite centers to promote protein intake and reduce sweet consumption [277]. Ketogenic diets enhance hepatic FGF-21 signaling, improving hepatic steatosis by promoting fatty acid oxidation and inhibiting lipogenesis [278]. Conversely, FGF-21 deficiency exacerbates liver damage in rodents fed high-fat, high-sugar, and alcohol-rich diets [279]. Diet al.so regulates MANF, which is positively correlated with body mass index (BMI) [280]. High-fat diets increase hepatic MANF release, activating the p38-MAPK pathway and promoting adipose tissue browning [280]. These findings suggest a role for MANF in the metabolic adaptation to dietary excess [280]. In addition, SHBG levels are influenced by macronutrient composition. High-fiber intake is positively associated with SHBG levels in older men, whereas high-protein diets have the opposite effect [281]. In postmenopausal women, combining a low-fat, high-fiber diet with aerobic exercise significantly elevates SHBG levels, highlighting the synergistic impact of dietary and exercise interventions on SHBG [282].

In contrast to FGF-21, MANF and SHBG can be modulated by macronutrient components in the diet, while research on Fetuin-A and SELENOP has focused on responses to specific dietary components. Consumption of coffee, dairy products, and alcohol lowers Fetuin-A levels, whereas high-fat diets elevate circulating Fetuin-A, promoting lipid-induced apoptosis and pancreatic β-cell damage [231, 283]. SELENOP levels are tightly regulated by selenium intake. Diets low in selenium reduce SELENOP levels, whereas selenium-rich foods, including fruits, vegetables, and antioxidant foods, increase its expression [284].

In summary, both exercise and diet are powerful modulators of hepatokine expression, offering therapeutic potential for metabolic disorders. Changes in hepatic factor levels induced by diet also have implications for CNS function. Future research could explore the synergistic effects of combined lifestyle interventions.

### Hepatokine-targeted therapeutics

Beyond lifestyle interventions like exercise and diet, several targeted therapies have been developed to correct hepatokine dysregulation. In cases of functional loss or downregulation of hepatokines, supplementing with recombinant proteins has proven effective. Structural modifications, including pegylation and glycosylation, enhance protein stability and prolong half-life, thereby improving therapeutic efficacy [285]. For instance, pegylated FGF-21 analogs have demonstrated promising results in treating NAFLD [279], while recombinant FGF-21 (rFGF-21) has demonstrated the ability to ameliorate BBB damage caused by T2DM [286]. Additionally, weekly injections of the Fc-FGF-21 analog AKR-001 have been found to significantly improve insulin sensitivity in patients with the T2DM, further supporting the clinical applicability of FGF-21-based therapies [287].

Monoclonal antibodies provide a powerful approach to counteract hepatokine overexpression. The ANGPTL3-neutralizing antibody Evinacumab has been proven to significantly reduce LDL cholesterol levels in patients with hypercholesterolemia, demonstrating its cardiovascular benefits [288]. Similarly, the SELENOP-neutralizing antibody AE2 not only reduces IR but also enhances insulin secretion [289]. These antibodies offer precise therapeutic modulation by targeting specific hepatokines involved in disease pathogenesis.

In addition to monoclonal antibodies, small molecule agonists or antagonists provide another strategy by modulating the receptor signaling pathways of hepatokines. NGM313, a novel activator of β-Klotho/FGFR1c, stimulates the FGF-21 signaling pathway to reduce body weight and hepatic fat accumulation [290]. Moreover, the bispecific antibody BFKB8488A operates through a similar pathway, thereby increasing serum adiponectin and decreasing both body weight and food intake [291]. In addition, the fibroblast activation protein (FAP) inhibitor BR103354 blocks FGF-21 degradation, significantly prolonging half-life of FGF-21 and improving metabolic phenotypes and hepatic steatosis in diabetic mice [292].

Gene therapy represents a cutting-edge approach to modulating hepatokines, though it remains largely experimental. Techniques such as adenoviral vector delivery and non-viral systems, including GalNAc-siRNA, have been explored for precise hepatokine regulation in target tissues [293]. FGF-21 overexpression in mouse models has been shown to maintain elevated FGF-21 levels leading to weight loss in obese mice [293]. Additionally, Vupanorsen, a GalNAc-modified antisense oligonucleotide (ASO) targeting ANGPTL3 mRNA, has demonstrated significant reductions in blood lipids and LDL cholesterol levels in clinical studies [294].

## Discussion and perspectives

This review highlights the intricate interplay between the liver and AD through the liver-brain axis, emphasizing its role in metabolic regulation, inflammatory signaling, and cognitive health. Hepatokines, such as FGF-21, SELENOP, Fetuin-A, and MANF, emerge as crucial mediators, influencing neurodegeneration by modulating insulin sensitivity, oxidative stress, lipid metabolism, and immune responses [13, 295]. Specific insights include the liver’s contribution to peripheral and hepatic clearance of Aβ, hepatic enzymes and metabolites associated with cognitive decline, and the systemic impact of metabolic disorders, such as IR and dyslipidemia, on AD progression. Furthermore, emerging evidence underscores the therapeutic and diagnostic potential of hepatokines in mitigating neurodegeneration. These findings collectively establish the liver as a critical interface in the systemic pathology of AD and a promising target for integrated therapeutic strategies.

Despite these advances, several critical challenges remain. Current understanding of the liver-brain axis in AD is largely derived from preclinical studies and observational data, leaving causal mechanisms underexplored [296]. Hepatokines exhibit dual roles in the brain and other organs – acting as both protective and harmful agents – therefore complicating their therapeutic application. Additionally, the interplay between genetic predisposition, such as ApoE ε4, and hepatic dysfunction in AD pathogenesis is insufficiently understood [297]. Moreover, while hepatokines show promise as diagnostic biomarkers, their specificity and sensitivity require validation in large-scale, longitudinal studies. The influence of confounding factors, such as age, sex, and comorbidities of NAFLD, further complicates the interpretation of findings. Lastly, therapeutic interventions targeting the liver-brain axis remain in the early stages, necessitating rigorous clinical trials to establish efficacy and safety.

Future research on the liver-brain axis should prioritize exploring the molecular mechanisms linking hepatic dysfunction to AD pathology, with a particular focus on the bidirectional effects of hepatokines on systemic and neural homeostasis. Advanced multi-omics approaches can provide deeper insights into these interactions, facilitating the development of high-sensitivity diagnostic methods to establish hepatokines as robust biomarkers for diagnosis and prognosis, validated through multi-center studies across diverse populations. More importantly, although we have collated the evidence on the penetration of hepatokines into the BBB, variations in research approaches and indirect measurements introduce uncertainties. Furthermore, given the prevalent BBB dysfunction in AD, the efficiency of hepatokines across the BBB may be significantly altered, impacting the therapeutic potential of these molecules. Furthermore, therapeutic strategies must integrate metabolic and neurodegenerative pathways, such as enhancing hepatic Aβ clearance through pharmacological or lifestyle interventions to slow AD progression. Additionally, investigating the liver’s role within the broader systemic context of diseases such as cardiovascular disease and diabetes, which share overlapping mechanisms with AD, may uncover common therapeutic targets and advance our understanding of this complex axis.

## Conclusion

The liver-brain axis offers a promising perspective on the systemic nature of AD. This review underscores the potential for integrated strategies targeting liver-brain crosstalk to mitigate neurodegeneration. Harnessing the therapeutic potential of hepatokines and addressing metabolic dysfunction may not only decelerate AD progression but also transform paradigms in neurodegenerative disease management. In this evolving landscape, the liver emerges not merely as a metabolic organ but as a critical player in the pathophysiology and therapy of AD.

**Table 1:** Clinical and Therapeutic Applications of Hepatokines

Hepatokine	Diagnostic Biomarkers	Exercise	Diet	Targeted Therapy
FGF-21	Lower levels in AD patients suggest a potential diagnostic role [201]	Enhances sensitivity, boosts fatty acid oxidation [204, 205]	Low-protein, ketogenic diets increase FGF-21 [213–215]	Recombinant FGF-21 analogs improve NAFLD and T2DM-related BBB damage [223–225]
SELENOP	Lack of research	HIIT increases SELENOP [209]	Selenium-rich diets boost SELENOP [221]	SELENOP-neutralizing antibodies reduce insulin resistance [227]
Fetuin-A	Inverse relationship with inflammatory markers; higher levels linked to better cognitive function [200]	Moderate exercise temporarily increases levels [210]	High-fat diets elevate levels; coffee and alcohol lower levels [219, 220]	Lack of research
ANGPTL3	Lack of research	Effects unclear	Effects unclear	ANGPTL3-neutralizing antibodies reduce LDL cholesterol [226, 233]
MANF	Overexpressed in AD patients’ temporal gyrus cortex; potential for early neurodegeneration detection [202]	Effects unclear	High-fat diets increase MANF [216]	Lack of research
Adropin	Lack of research	Effects unclear	Effects unclear	Lack of research
ApoJ	Shows promise in predicting progression from MCI to AD [117]	Exercise lowers levels [212]	Effects unclear	Lack of research
SHBG	Elevated SHBG levels correlate with AD risk in older populations [199]	Effects unclear	High-fiber diets increase SHBG [217]	Lack of research

## Electronic supplementary material

Below is the link to the electronic supplementary material.

Supplementary Material 1

## Data Availability

Not applicable.

## Abbreviations

AAA: Aromatic Amino Acids
ACC: Acetyl-Coa Carboxylase
AD: Alzheimer’s Disease
ALP: Alkaline Phosphatase
ALS: Amyotrophic Lateral Sclerosis
ALT: Alanine Aminotransferase
ANGPTL3: Angiopoietin-Like Protein 3
ApoA-I: Apolipoprotein A-I
ApoB: Apolipoprotein B
ApoE: Apolipoprotein E
ApoJ: Apolipoprotein J
AREs: Antioxidant Response Elements
ASO: Antisense Oligonucleotide
AST: Aspartate Transaminase
ATF2: Activating Transcription Factor 2
ATF4: Activating Transcription Factor 4
ATF5: Activating Transcription Factor 5
ATF6: Activating Transcription Factor 6
α-syn: α-synuclein
Aβ: β-amyloid
BAT: Brown Adipose Tissue
BBB: Blood-Brain Barrier
BCAAs: Branched-Chain Amino Acids
BDNF: Brain-Derived Neurotrophic Factor
BMI: Body Mass Index
CET: Continuous Exercise Training
ChREBP: Carbohydrate Response Element-Binding Protein
CNS: Central Nervous System
CSF: Cerebrospinal Fluid
CEC: Cholesterol Efflux Capacity
ER: Endoplasmic Reticulum
EDSS: Expanded Disability Status Scale
FAP: Fibroblast Activation Protein
FCS: Fluorescence Correlation Spectroscopy
FFAR4: Free Fatty Acid Receptor 4
FGF: Fibroblast Growth Factor
fMRI: Functional Magnetic Resonance Imaging
FST: Follistatin
GGT: Gamma-Glutamyl Transferase
GRP78: Glucose-Regulated Protein 78
Gpx3: Glutathione Peroxidase 3
Gpx4: Glutathione Peroxidase 4
GWAS: Genome-Wide Association Studies
HDL: High-Density Lipoprotein
HIIT: High-Intensity Interval Training
HMGB1: High-Mobility Group Box 1
Hnf4α: Hepatic Nuclear Factor 4 Alpha
HSA: Human Serum Albumin
IR: Insulin Resistance
LDL: Low-Density Lipoprotein
LDL-C: Low-Density Lipoprotein Cholesterol
LPL: Lipoprotein Lipase
LRP-1: LDL Receptor-Related Protein 1
MANF: Midbrain Astrocyte-Derived Neurotrophic Factor
MASLD: Metabolic-Associated Fatty Liver Disease
MCI: Mild Cognitive Impairment
MMSE: Mini-Mental State Examination
MoCA: Montreal Cognitive Assessment
MS: Multiple Sclerosis
NAFLD: Nonalcoholic Fatty Liver Disease
NF-κB: Nuclear Factor Κ-Light-Chain-Enhancer of Activated B Cells
NSC: Neural Stem Cell
PD: Parkinson’s Disease
PDHC: Pyruvate Dehydrogenase Complex
PET: positron emission computed tomography
PUFAs: Polyunsaturated Fatty Acids
rFGF-21: Recombinant FGF-21
ROS: Reactive Oxygen Species
Se: Selenium
Sec: Selenocysteine
SELENOP: Seleno-protein P
SelP-H: histidine-rich domain of SELENOP
SHBG: Sex Hormone-Binding Globulin
SMOC1: Sparc-Related Modular Calcium-Binding Protein 1
sEH: Soluble Epoxide Hydrolase
TG: Triglyceride
TSK: Tsukushi
T2DM: Type 2 Diabetes Mellitus
UPR: Unfolded Protein Response
VLDL: Very Low-Density Lipoprotein
