# Supplementary material for: Bridging systemic metabolic dysfunction and Alzheimer’s disease: the liver interface
Source: Mol Neurodegener. 2025 May 28;20:61. doi: 10.1186/s13024-025-00849-6 (PMC12121119; doi:10.1186/s13024-025-00849-6)
Supplement: Supplementary file 1 — Supplementary Material 1 [file 13024_2025_849_MOESM1_ESM.docx]

December 19, 2024

Dear editor in chief Guojun Bu, PhD

We are submitting the enclosed manuscript entitled “***Bridging Systemic Metabolic Dysfunction and Alzheimer’s Disease: The Liver Interface***” for your consideration as a review article in ***Molecular Neurodegeneration***.

This review synthesizes emerging insights into the liver’s pivotal role in Alzheimer’s disease (AD) pathogenesis through systemic metabolic regulation and the liver-brain axis, offering a fresh perspective on this increasingly recognized interconnection. The review addresses the pressing need to understand AD as a systemic disorder, highlighting how hepatic mechanisms—such as Aβ clearance, hepatokine signaling, and metabolic dysfunction—interface with core neuropathological features, including Aβ aggregation, Tau hyperphosphorylation, neuroinflammation, and oxidative stress. Notably, we emphasize the roles of hepatokines, such as FGF-21, SELENOP, and Fetuin-A, as mediators of liver-brain crosstalk. These proteins represent potential biomarkers and therapeutic targets, connecting systemic metabolic health with cognitive decline.

What sets this review apart is its integrated focus on the liver as both a systemic regulator and a therapeutic entry point for AD. We extend the discussion beyond molecular mechanisms to propose actionable strategies, such as lifestyle interventions, that regulate hepatokine expression and offer innovative approaches for prevention and treatment. By presenting the liver as a hub bridging metabolic dysfunction with neurodegeneration, our work advances a holistic framework that aligns with contemporary efforts to reframe AD as a systemic disorder.

We believe our work aligns well with the scope and interests of ***Molecular Neurodegeneration*** and will capture significant interest among its readers.

I would like to declare on behalf of my co-authors that the manuscript has not been previously published and is not under consideration elsewhere. All authors have reviewed and approved the manuscript. and we have no conflicts of interest to disclose. We look forward to your feedback and hope for the opportunity to contribute to the ongoing research in this important area.

Thank you for your time and consideration.

Best regards,

**Xiu-Qing Yao**, M.D, Ph.D.

Department of Rehabilitation, The Second Affiliated Hospital of Chongqing Medical University, Chongqing, China.

**Tel:** +86 (23) 63693883.

**Email:** dryaoxq@cqmu.edu.cn
